# Responsive manganese-based nanoplatform amplifying cGAS-STING activation for immunotherapy

**DOI:** 10.1186/s40824-023-00374-x

**Published:** 2023-04-15

**Authors:** Qingbin He, Runxiao Zheng, Junchi Ma, Luyang Zhao, Yafang Shi, Jianfeng Qiu

**Affiliations:** 1 School of Radiology, Shandong First Medical University & Shandong Academy of Medical Sciences, Tai’an, 271000 China; 2 Medical Science and Technology Innovation Center, Shandong First Medical University & Shandong Academy of Medical Sciences, Jinan, 250000 China; 3 School of Radiology, the Second Affiliated Hospital of Shandong First Medical University, Tai’an, 271016 China

**Keywords:** Tumor responsiveness, cGAS-STING signaling pathway, Immunotherapy, Manganese ions, Reactive oxygen species

## Abstract

**Background:**

The activation of the cyclic guanosine monophosphate-adenosine monophosphate synthase-stimulator of interferon genes (cGAS-STING) signaling pathway has attracted great attention for its ability to up-regulate innate immune response and thus enhance cancer immunotherapy. However, many STING agonists limit the further advancement of immunotherapy due to weak tumor responsiveness or low activation efficiency. The responsive and effective activation of cGAS-STING signaling in tumors is a highly challenging process.

**Methods:**

In this study, a manganese-based nanoplatform (MPCZ NPs) was constructed that could responsively and efficiently generate more manganese ions (Mn^2+^) and reactive oxygen species (ROS) to activate cGAS-STING signaling pathway. Briefly, manganese dioxide (MnO_2_) was loaded with zinc protoporphyrin IX (ZPP) molecule and coated by polydopamine (PDA) embedded with NH_4_HCO_3_ to obtain MPCZ NPs. Additionally, MPCZ NPs were evaluated in vitro and in vivo for their antitumor effects by methyl thiazolyl tetrazolium (MTT) assay and TUNEL assays, respectively.

**Results:**

In this system, tumor responsiveness was achieved by exogenous (laser irradiation) and endogenous (high levels GSH) stimulation, which triggered the collapse or degradation of PDA and MnO_2_. Moreover, the release of Mn^2+^ augmented the cGAS-STING signaling pathway and enhanced the conversion of hydrogen peroxide (H_2_O_2_) to hydroxyl radical (·OH) under NIR laser irradiation. Furthermore, the release of ZPP and the elimination of GSH by MPCZ NPs inhibited HO-1 activity and prevented ROS consumption, respectively.

**Conclusions:**

This adopted open source and reduce expenditure strategy to effectively generate more ROS and Mn^2+^ to responsively activate cGAS-STING signaling pathway, providing a new strategy for improving immunotherapy.

**Supplementary Information:**

The online version contains supplementary material available at 10.1186/s40824-023-00374-x.

## Background

Immunotherapy, which can attack the tumor by harnessing the host immune system, has become one of the main cancer treatments in the past two decades [1]. Nevertheless, only 20% of patients respond to immunotherapy such as chimeric antigen receptor T-cell immunotherapy (CAR-T therapy) or immune checkpoint blockade (ICB) and so on, which may be due to weak tumor immunogenicity at the lesion site or significant individual differences [2–4]. It has been reported that activation of innate immunity and cascade initiation of acquired immunity are important strategies to enhance tumor immunogenicity and reduce the limitations of individual differences [5]. In addition, tumor sites often lack sufficient innate immune responses, which not only prevent adaptive immune cells from recognizing tumors, but further promote tumor metastasis and recurrence as well [6, 7]. Besides, relatively few patients benefit from acquired immunotherapy alone [8]. Consequently, how to activate innate immunity is a crucial step for immunotherapy improvement.

Recently, a promising and popular approach has been developed to trigger innate immunity and drive anti-tumor adaptive immunity by activating the cyclic guanosine monophosphate-adenosine monophosphate (GMP-AMP) synthase-stimulator of interferon genes (cGAS-STING) signaling pathway and releasing type I interferons (IFNs) [9]. Damaged intracellular DNA is sensed by GMP-AMP synthase (cGAS) [10, 11], which generates cyclic GMP-AMP from adenosine triphosphate (ATP) and guanosine triphosphate (GTP), activating STING and promoting IFN production [12–14]. Type I IFN signaling enhances tumor-specific antigen presentation on dendritic cells (DCs) and cross-progenitor anti-tumor T cells for adaptive immunity [15, 16]. Nevertheless, single drugs that rely solely on STING agonists, such as cyclic dinucleotide (CDN), are not significantly effective in two phase I clinical trials against solid tumors or lymphomas [17], which could attribute to rapid clearance and poor internalization of STING agonists [18] and immunosuppression of excessive STING agonists [19]. Hence, how to activate efficiently and responsively cGAS-STING signals in tumors remains a thorny problem.

Nanotechnology is one of the major technologies in the future field of science [20]. Nanomaterials have the advantages of targeting tumor tissue, responsively releasing drugs and superior optical, magnetic and other properties [21, 22]. Thus, high-efficiency and responsive activation of STING signaling pathway by nanomaterials is an effective way to amplify innate immunity. Considering that the activation of cGAS-STING signaling pathway originates from the leakage of double-stranded DNA (dsDNA) into the cytoplasm, the generation of excess intracellular reactive oxygen species (ROS) can be a strategy to damage mitochondria and release mitochondrial DNA [3, 23, 24]. To significantly improve ROS generation efficiency, open source and reduce expenditure strategy was effective for multi-level ROS generation [25]. In addition, metal ions play an amazing role in immune regulation [8]. As an example, manganese ions (Mn^2+^) have been reported to activate cGAS-STING signaling through inducing the phosphorylation of TBK1 and p65. Recently, it has been reported that Mn^2+^ can also improve the catalytic activity of cGAS, STING activity and cGAMP-STING affinity [26]. Therefore, the rational design of nanomaterials can make them responsive to produce abundant ROS and Mn^2+^, which can effectively enhance STING signaling pathway activation and amplify innate immunity.

**Fig. 1:**
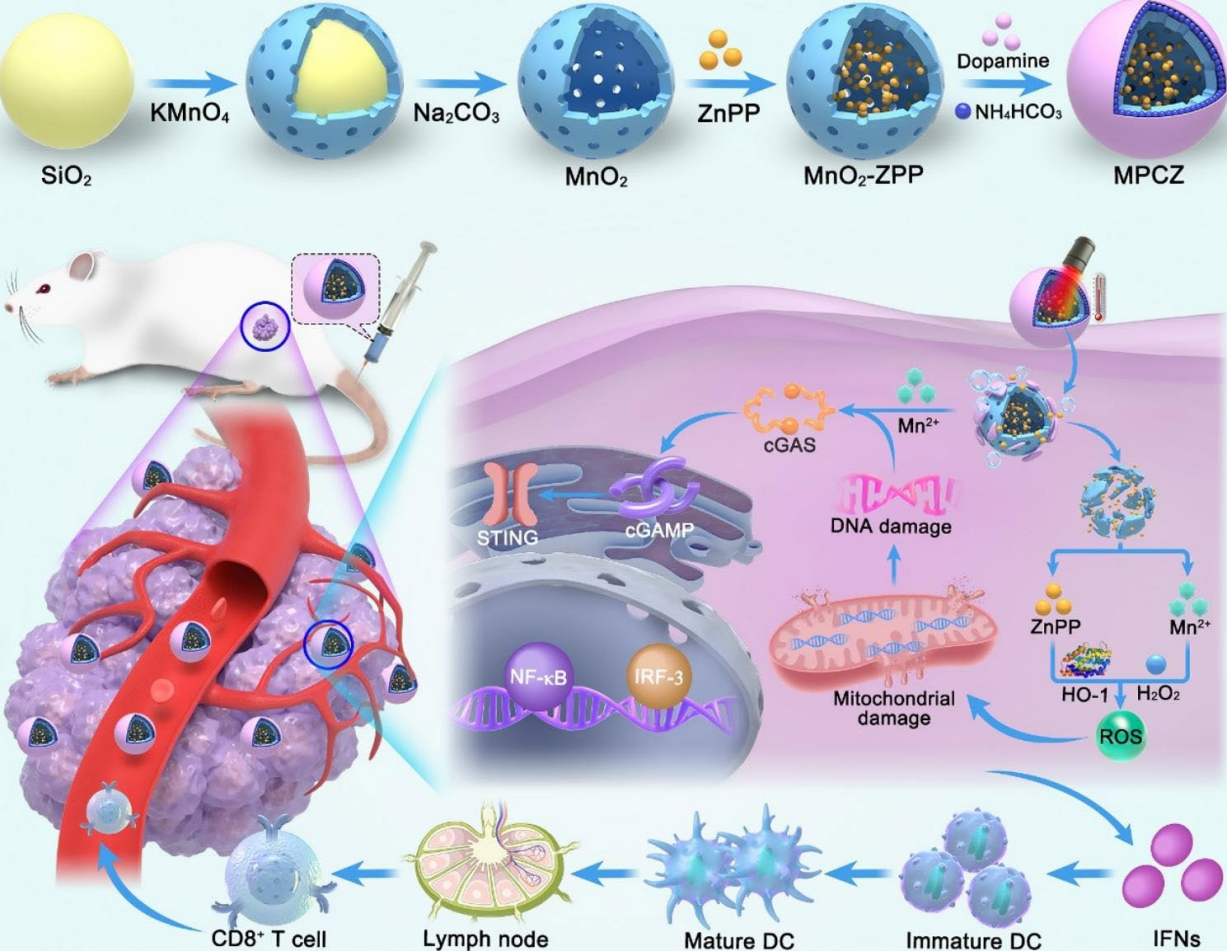
Schematic illustration of MPCZ nanoplatforms as ROS generation and Mn^2+^ release for enhanced STING signaling pathway activation and amplified innate immunotherapy. After MPCZ NPs reaches the tumor area, the PTT effect of PDA can promote the decomposition of NH_4_HCO_3_, the collapse of PDA and the exposure of MnO_2_ under 808 nm laser irradiation. Moreover, in tumor cells, under the action of high concentration GSH, the generation of Mn^2+^ and the release of ZPP will be triggered, and more ROS will be generated in an open source and reduce expenditure strategy. The generation of ROS and Mn^2+^ can jointly promote the activation of cGAS-STING signaling pathway and enhance the anti-tumor immune response

In this study, dual-responsive manganese-based nanoplatform (MPCZ NPs) was constructed to amplify cGAS-STING activation for cancer innate immunotherapy. As illustrated in Fig. 1, hollow MnO_2_ nanoparticles (hMnO_2_ NPs) was loaded with zinc protoporphyrin IX (ZPP) and coated by polydopamine (PDA) embedded with NH_4_HCO_3_ to obtain MPCZ NPs. First, MPCZ NPs accumulate in the tumor sites through enhanced permeability and retention (EPR) effect. Then, the internal NH_4_HCO_3_ could be triggered by the external responsive stimulus (an 808 nm laser) to produce CO_2_ and NH_3_ due to the photothermal effect, which would cause the PDA to break and release ZPP. Subsequently, exposed hMnO_2_ NPs will collapse and degrade further under the reducing action of high level GSH (endogenous responsive stimulus) in tumor cells. The release of Mn^2+^ can not only activate the STING signaling pathway, but also exert the chemodynamic therapy (CDT) effect and generate abundant ROS under the action of high level H_2_O_2_ in tumor cells. Moreover, the photothermal therapy (PTT) effect of PDA will further promote the production of ROS, acting as the opening source strategy for ROS generation. Besides, the collapse of hMnO_2_ further promotes the release of ZPP, which interacts with heme oxygenase (HO-1) by competitively with heme to inhibit the antioxidant activity of tumor cells, and the consumption of GSH also reduces the loss of generated ROS, terming reduce expenditure. This open source and reduce expenditure strategy are adopted to efficiently generate more ROS. Therefore, exogenous laser irradiation and endogenous high level GSH can be used to turn on MPCZ NPs in a responsive manner to generate abundant ROS and Mn^2+^ in tumor cells, thus efficiently and responsively to activate cGAS-STING signaling pathway and amplify innate immunotherapy.

## Methods

### Materials

Tetraethoxysilane (TEOS) is available from Sigma-Aldrich. Manganese permanganate (KMnO_4_), Na_2_CO_3_, ammonia, H_2_O_2_ solution (w/w: 30%) were brought from Sinopharm Chemical Reagent Co., Ltd. (China). Dopamine hydrochloride, methylene blue (MB) and ammonium bicarbonate (NH_4_HCO_3_) were provided by Aladdin (China). All experiments and buffer preparations were done using DI water. No further purification was performed on any of the reagents.

### Synthesis of MPCZ NPs

#### Synthesis of hMnO_2_ NPs

A modified Stöber method was used to synthesize silica nanospheres. An experiment was carried out in a beaker by adding ethanol (150 mL), ammonia (3 mL, 28%) and deionized (DI) water (30 mL) together. TEOS (12 mL) was added to the mixture while it was stirring, and continued for 12 h. The suspension turned murky white. The products were three times washed with water and ethanol after centrifugation at 10,000 g for 10 min. In 60 mL of DI water, the product was resuspended, and 2 g of potassium permanganate was added. An autoclave lined with Teflon was used to sterilize the suspension after sonicating it for 30 min. Heat was applied to the suspension for 48 h in an oven set to 150 °C. Following 15 min of centrifugation at 15,000 rpm, the precipitate was dispersed in Na_2_CO_3_ (2 M) solution at 60 °C for 12 h. A centrifuge at 6,000 g for 20 min was used to obtain hMnO_2_ NPs, which were washed with water twice before use.

#### Synthesis of MPCZ NPs

The hMnO_2_ NPs were dispersed in DMSO solution containing ZPP (1 mg/mL), stirred for 18 h at 30 °C and collected by centrifugation at 8,000 g for 10 min. Dopamine hydrochloride (15 mg), NH_4_HCO_3_ (20 mg) and MnO_2_-ZnPP (10 mg) were redispersed in Tris-HCl buffer (20 mL, 10 mM, pH 8.5). After the mixture was continued stirred at 30 °C for 1d, MPCZ NPs were obtained by centrifuging at 8,000 g for 20 min and washing with DI water. Similar protocols were used to prepare PDA and NH_4_HCO_3_ coated MnO_2_ (MPC NPs).

### Characterization of nanoparticles

Transmission electron microscopy (TEM) images were performed at an 80 kV acceleration voltage by HT7800. A TESCAN MIRA LMS transmission electron microscope was used to take Scanning Electron Microscope (SEM) images. The concentration of ZPP were measured by a double-beam UV-spectrophotometer (UV-8000 A, Shanghai, China) and high-pressure liquid chromatography (HPLC). Fourier Transform Infrared Spectroscopy (FTIR) measurements were carried out on PDA with a Thermo Scientific Nicolet iS20 (Thermo Fisher Scientific, USA) at 4000 – 400 cm^− 1^. Crystal structure measurements were performed with an Ultima IV X-ray diffractometer (XRD, Ultima IV, Rigaku, Japan) at 10°/min using Cu K_α_ radiation (λ = 1.54 Å). By using an ASAP2460, the Brunauer-Emmett-Teller (BET) surface area of hMnO_2_ was determined at 77 K by analyzing N_2_ adsorption-desorption isotherms. X-ray photoelectron spectroscopy (XPS) was also conducted on MnO_2_ using a Thermo Scientific K-alpha X-ray Photoelectron Spectrometer System (h*v* = 1486.68 eV). The hydrated particle size and zeta potential of the nanoparticles were determined by Dynamic Light Scattering (DLS, Malvern Zetasizer Nano ZS90).

### Measurement of photothermal performance

In a centrifuge tube, 0.5 mL of H_2_O, 400 µg/mL hMnO_2_ and MPCZ (100, 200 and 400 µg/mL) solution were irradiated by a NIR laser at 808 nm for 10 min at 1 W/cm^2^. Throughout the experiment, a thermocouple (the accuracy of ± 0.1 ºC) was immersed in the solution and the temperature of the solution was recorded every 30 s. Avoid direct laser light reaching the thermocouples by placing them directly above them. Infrared Thermal Imager (FLIR, USA) was used to photograph the infrared thermal images every 100 s.

### Observation of gas generation

A microscope slide was coated with a coverslip covering 30 µL of MPCZ (50 µg/mL) and irradiated with an NIR laser (1 W/cm^2^) for 10 min. The control group was the one that was not irradiated. Finally, the slide was inspected with a microscope with a 20 × objective.

### Measurement of GSH depletion

The depletion of GSH by nanoparticles was determined via Ellman’s assay. A mixture of 0.5 mL MPCZ solution (12.5, 25, 50, 100 µg/mL) and 0.5 mL of GSH solution (1 mM) was irradiated by a NIR laser (1 W/cm^2^, 10 min) before incubating at 37°C for 30 min. The supernatant of the solution was obtained by centrifugation at 8,000 g for 10 min. 1.26 mL Tris-HCl (pH = 8.0, 0.05 M) buffer and 10 µL 5,5’-dithioic-(2-nitrobenzoic acid) (DTNB, 100 mM, DMSO) were added to the supernatant, and then the absorption values at 410 nm were evaluated using UV-vis absorption spectrometry.

### Drug and Mn^2+^ release

MPCZ NPs was added in PBS buffer (pH = 6.5) with the presence or absence of GSH (10 mM), and irradiated with NIR laser (1 W/cm^2^, 10 min) or not, respectively. An incubation water bath was used to incubate solutions at 37 °C with slow magnetic stirring (200 rpm). Supernatants were collected by centrifugation at 8,000 g for 10 min at scheduled intervals, and precipitate was re-dispersed in PBS buffer. Inductively coupled plasma mass spectrometry (ICP-MS) was used to calculate the ion release rate at various times.

### Measurement of Mn^2+^-mediated Fenton reactions

In a buffer solution with 25 mM NaHCO_3_/CO_2_, 10 mg/mL MB, 8 mM H_2_O_2_, and 0.5 mM MnCl_2_ were allowed to react at 37 ºC for 30 min. The change in absorbance at 665 nm was used to track MB deterioration brought on by ·OH. For the MPCZ NPs, various concentrations of GSH (0, 1, 5, and 20 mM) were added to a NaHCO_3_ buffer solution (25 mM) containing MPCZ NPs (comparable to 200 µg/mL MPC or 10.0 µg/mL ZPP). After NIR laser irrigation (10 min) and shaking (5 min), the supernatant was obtained by centrifugation at 8,000 g for 10 min, and MB (10 µg/mL) and H_2_O_2_ (8 mM) were added. After 30 min of incubation at 37 °C, MB was measured at 665 nm for the change in absorbance.

### Cell culture

4T1 cells was cultured in Dulbecco’s modified Eagle’s medium (DMEM) with 10% FBS, 1% penicillin and 1% streptomycin at 37 ºC in a 5% CO_2_ atmosphere. Every two days, the medium was changed, and the cells were pass through trypsin before fusing.

### In vitro anticancer therapy

4T1 cells were plated into 96-well plates and incubated for 16 h in a cell incubator. Consequently, the culture medium was changed by fresh medium which contained MPC and MPCZ NPs (comparable to 25, 50, 100 and 200 µg/mL MPC or 1.25, 2.5, 5.0 and 10.0 µg/mL ZPP) in normal culture medium. After cultured for 6 h, 4T1 cells were irradiated with or without NIR laser (1 W/cm^2^, 10 min) and cultured for the following 18 h. Then methyl thiazolyl tetrazolium (MTT) assay was performed to evaluate the cell viability. For assessment of Live/dead staining, the procedure for treating 4T1 cells with nanodrugs is similar to that in MTT assay. Then cells were stained with propidium iodide (PI) and calcein AM for 30 min and observed by a fluorescence microscope.

### Cell apoptosis measurement

4T1 cells were plated into 6-well plates with a density of 1.6 × 10^5^ cells/well and incubated for 16 h in a cell incubator. Then, cells were cultured with medium containing PBS, MPC and MPCZ NPs (comparable to 100 µg/mL MPC or 5.0 µg/mL ZPP) for 6 h and irradiated by NIR laser (1 W/cm^2^, 10 min) for following 18 h incubation. To confirm apoptosis, the Annexin V-FITC/PI apoptosis detection kit was used combined with flow cytometry.

### Western blot analysis

With the density of 1.6 × 10^5^ cells/well, 4T1 cells were transferred into 6-well plates and cultured for a full night at 37 ºC in a cell incubator. Then, cells were incubated with PBS, MPC, MPCZ NPs (comparable to 25 µg/mL MPC or 1.25 µg/mL ZPP) for 6 h and irradiated with or without NIR laser (1 W/cm^2^) for 10 min following 6 h incubation. As previously reported in the literature [27], after the cells were lysed, we separated same amounts of proteins using sodium dodecyl sulfate polyacrylamide gel electrophoresis (SDS-PAGE) and transferred them to polyvinylidene difluoride (PVDF) membranes. We performed western blotting analysis using PVDF membranes incubated with HO-1, STING, p-SING, TBK1 and p-TBK1.

### IFN-β and CXCL10 accessed by ELISA

4T1 cells were plated into 96-well plates with a density of 1.0 × 10^4^ cells/well and incubated 12 h at 37 ºC in a 5% CO_2_ atmosphere. Then, the cells were incubated with PBS or MPC or MPCZ NPs (comparable to 25 µg/mL MPC or 1.25 µg/mL ZPP) for 6 h, irradiated with or without NIR laser (1 W/cm^2^) for 10 min, and incubated for another 6 h. Then, the cell debris and nanoparticles were centrifuged at 2000 g for 10 min at room temperature from the culture plate and 50 µL supernatant was obtained. IFN-β and CXCL10 cytokines were detected by ELISA kit according to the instructions [18].

### The maturation of DCs in vitro

Firstly, the Bone-marrow-derived dendritic cells (BMDCs) were extracted from 8 to 10 weeks old BALB/c mouse thighbone. To evaluate the BMDCs’ maturation, DCs were plated in the bottom of the transwell system, while 4T1 cells were put in the upper compartment incubated by PBS, MPC, MPCZ NPs (comparable to 25 µg/mL MPC or 1.25 µg/mL ZPP) for 6 h, irradiated with or without NIR laser (1 W/cm^2^) for 10 min, and incubated for another 6 h. Then, DCs were collected and stained with APC-CD11c, FITC-CD80, and PE-CD86 monoclonal antibodies followed by flow cytometry analysis.

### Measurement of HO-1 activity in 4T1 cells

In six-well plates, 4T1 cells (1.6 × 10^5^) and normal culture medium (1.6 mL) was added to each well overnight for growth. Then, 4T1 cells were incubated with fresh normal culture medium containing MPC and MPCZ NPs (comparable to 25 µg/mL MPC or 1.25 µg/mL ZPP) for 6 h, and NIR laser (1 W/cm^2^) was used to irradiate the cells. After incubated for the following 6 h, then cells were homogenized and centrifuged at 10,000 g for 30 min at 4 °C, mixed 10 µM hemin, 1 mg cytosolic fraction of rat liver and 0.8 mM nicotinamide adenine dinucleotide phosphate in 1 mL PBS. After 60 min of incubation in the dark at 37 °C, 1 mL of trichloromethane was added to terminate the reaction. Bilirubin concentration was determined using the difference between absorbance at 465 and 530 nm.

### Intracellular GSH depletion

In six-well plates, 4T1 cells (1.6 × 10^5^) and normal culture medium (1.6 mL) was added to each well overnight for growth. In the following steps, cells were cultured with normal culture medium containing MPC and MPCZ NPs (comparable to 25 µg/mL MPC or 1.25 µg/mL ZPP) with normal culture medium for 6 h, then irradiated with or without NIR laser for 10 min and incubated for another 6 h. After lysis of the cells, the mixture of 30 µL cell supernatant and 150 µL 30 µg/mL DTNB incubated for 25 min. Finally, SpectraMax M3 microplate reader was used to detect 412 nm absorption values, which were positively correlated with GSH levels.

### The detection of intracellular ROS and mitochondrial membrane potential

DCFH-DA and JC-1 were used to detect intracellular ROS and mitochondrial membrane potential. First, 4T1 cells were plated in 6-well plate with normal culture medium for 12 h. Then incubation of cells with fresh normal culture medium containing MPC and MPCZ NPs (comparable to 25 µg/mL MPC or 1.25 µg/mL ZPP) for 6 h was followed by exposure to the NIR laser (1 W/cm^2^) for 10 min and incubation for another 6 h. Finally, cells were incubated with DCFH-DA or JC-1 in fresh medium for 30 min. For JC-1 assay, the cell nucleus was stained with DAPI. The fluorescence images were observed with a fluorescence microscope. Moreover, the fluorescence intensity of intracellular DCF was also quantitatively analyzed by flow cytometry.

### The 4T1 tumor-bearing mouse model establishment and treatments

To explore the anti-tumor immune effect of MPCZ NPs in vivo, antitumor studies were conducted using the 4T1 tumor-bearing mice model. As a method of tumor inoculation, 100 µL of PBS containing 5 × 10^7^ 4T1 cells was injected into the back of female Balb/c mice. After 7 d, the 4T1 tumor-bearing mice were randomly divided randomly into six groups: PBS, MPC, MPCZ, PBS plus NIR, MPC plus NIR, and MPCZ plus NIR group. On day 0 and day 4, PBS, MPC and MPCZ (comparable to 20 mg/kg mouse for MPC and 1 mg/kg mouse for ZPP) were injected by tail vein. After 24 h of injection, NIR (1 W/cm^2^) laser was irradiated for 10 min. To study the immune effect of NPs, BALB/c mice were subcutaneously injected with 5 × 10^7^ 4T1 cells into the left flank (primary tumor), and with 5 × 10^6^ 4T1 cells into the right flank (abscopal tumor). Then six groups of the 4T1 tumor-bearing mice were injected by tail vein on day 0 and day 4. After 24 h of injection, NIR (1 W/cm^2^) laser was irradiated primary tumor for 10 min. Abscopal tumor was without NIR laser irradiation. Over the course of the experiment, body weight and tumor volume were recorded, with tumor volume being calculated by (length × width × width)/2. The mice were sacrificed on the 15th day, and the tumors, heart, liver, spleen, lung, and kidney were stained with H&E.

### Hemolysis Test

Red blood cells (from mouse blood) were washed with PBS to remove serum, and then suspended in PBS (20 mL). MPCZ NPs was added to the red blood cell suspension (comparable to 25, 50, 100, 200, 400 and 800 µg/mL MPC or 1.25, 2.5, 5.0, 10.0, 20.0 and 40.0 µg/mL ZPP). Red blood cell suspensions diluted in DI water were positive controls, while those diluted in PBS were negative controls. Based on the previous description, we measured and calculated the hemolysis rate after the reaction was conducted at 37 ºC for 3 h [28].

### In vivo fluorescence imaging

BALB/c mice were subcutaneously inoculated with 100 µL of PBS containing 5 × 10^7^ 4T1 cells at the back. When the tumor volume reached approximately 150 mm^3^, mice were intravenously administered with 100 µL of free Cy5 and Cy5-loaded MPCZ NPs (equivalent to 0.5 mg Cy5/kg mouse). Then, the mice were euthanized for fluorescence imaging.

### H&E, TUNEL assay and immunofluorescence staining

Paraformaldehyde 4% was used to fix the tumor tissue, which was then embedded in paraffin. Tissue sections were treated with H&E staining, cell morphology of each group was observed, and pathological changes were analyzed. Apoptosis was detected by terminal deoxynucleotidyl transferase-mediated dUTP nick-end labeling (TUNEL) assay. For immunofluorescence staining, Tissue sections were incubated with FITC-antimouse CD80 antibody, and PE antimouse CD86 antibody and stained with DAPI.

### The evaluation of immune system activation in vivo

The 4T1 tumor-bearing mice were euthanized to obtain tumor-draining lymph nodes and spleen on day 8 for flow cytometry analysis. To obtain suspended single cells, lymph nodes and spleen were cut into small pieces and homogenized. To evaluate DCs maturation of tumor-draining lymph nodes in vivo, APC-CD11c, FITC-CD80, and PE-CD86 monoclonal antibodies were used to stain single cells of tumor-draining lymph nodes. Moreover, the immune cells in the tumor-draining lymph node and spleen were stained with APC-CD3, FITC-CD4, and PE-CD8a to analyze the proportion of activated CD4^+^ helper T cells and CD8^+^ T cells.

### Statistical analysis

All data was presented as mean or means ± standard deviation. A two-tailed Student’s t-test was used to calculate the significance of the comparisons between two groups. For multiple comparisons, one- or two-way ANOVA with Tukey’s or Sidak’s multiple comparisons test was adopted by GraphPad Prism. P < 0.05 was considered as statistically significant.

## Results

### Synthesis and characterization of MPCZ NPs

**Fig. 2:**
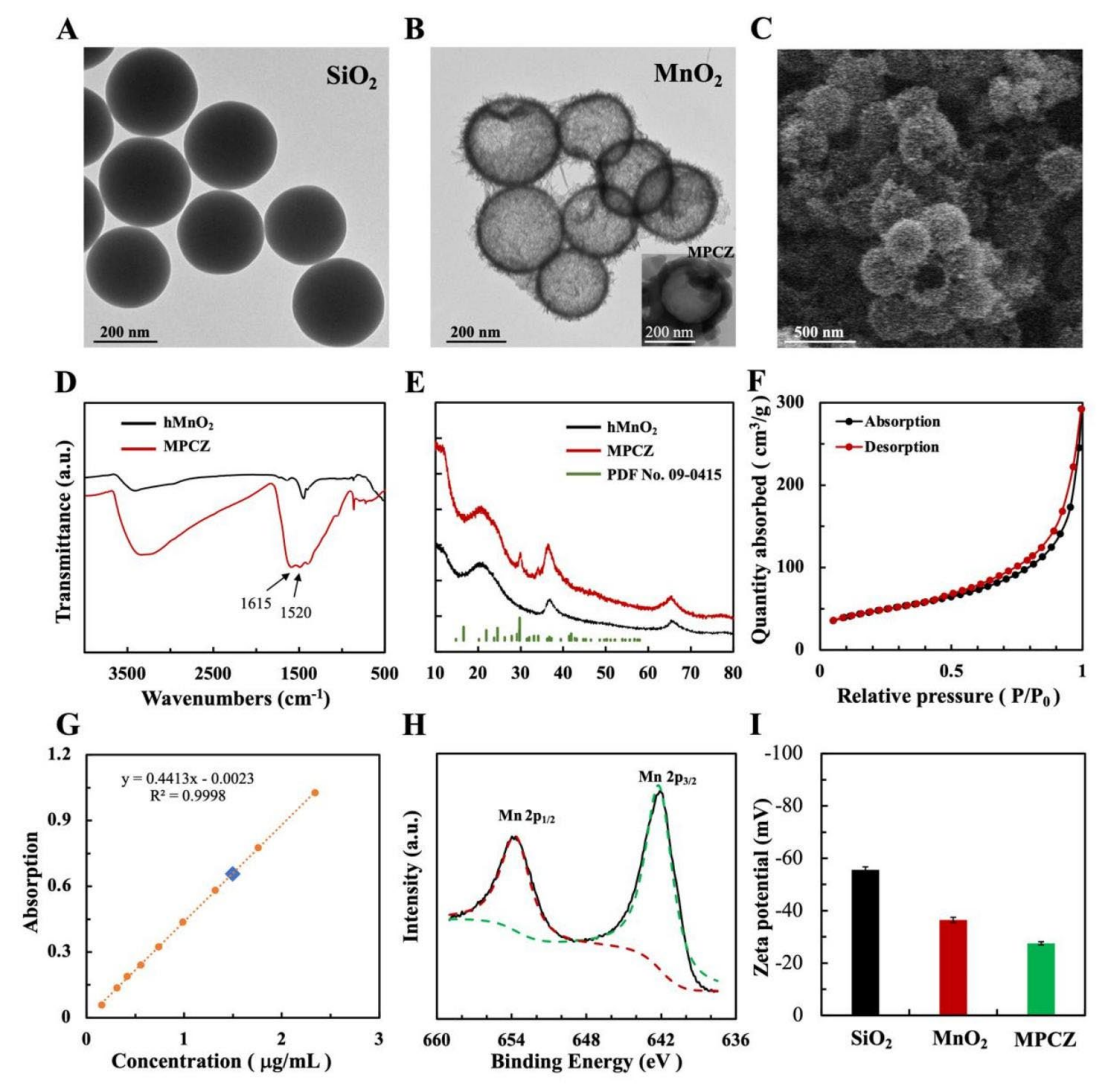
**Synthesis and characterization of MPCZ NPs.** TEM images of (**A**) SiO_2_ and (**B**) hMnO_2_. The insert of (**B**) is TEM image of MPCZ NPs. (**C**) SEM image of hMnO_2_. FTIR spectrum (**D**) and XRD patterns (**E**) of MPCZ NPs (NH_4_HCO_3_ crystals have an orthorhombic structure (PDF No.09-0415)). (**F**) N_2_ adsorption/desorption isotherms of hMnO_2_. (**G**) UV–vis spectroscopy calibration curve for measuring ZPP loading efficiency. (**H**) XPS spectra of Mn 2p region of hMnO_2_. (**I**) Zeta potentials of SiO_2_, hMnO_2_ and MPCZ NPs during the preparation process

The synthetic procedure of the MPCZ NPs is illustrated in Fig. 1. First, TEOS was hydrolyzed to produce monodisperse silica nanoparticles (SiO_2_ NPs) that were used as a hard template immediately [29] (Fig. 2A). In order to cover the surface of SiO_2_ NPs with MnO_2_, KMnO_4_ was mixed with SiO_2_ NPs, and the KMnO_4_ was reduced by unreacted organosilica. Sodium carbonate solution was used to etch silica from MnO_2_@SiO_2_ nanoparticles in order to obtain hMnO_2_ NPs. According to TEM and SEM images (Fig. 2B C), hMnO_2_ NPs were spherical and uniform in size, with a hollow structure. Finally, hMnO_2_ NPs loaded ZPP and coated PDA embedded with NH_4_HCO_3_ to enhance physiological stability and reduce leakage. According to the TEM image (inset of Fig. 2B), the PDA shells of the MPCZ NPs could be clearly seen nearly 30 nm. Moreover, FTIR spectrum was collected to further confirm PDA development as shown in Fig. 2D. It was evident that hMnO_2_ NPs have successfully been coated with PDA because their absorption peaks located in 1520 and 1615 cm^− 1^, respectively, corresponding to shearing vibrations of N-H in the amide group and aromatic rings [30]. In addition, XRD patterns revealed the incorporation of NH_4_HCO_3_. In the Fig. 2E, MPCZ NPs exhibited an additional peak located at 29.1° in the XRD pattern compared with hMnO_2_ NPs, which has been assigned to NH_4_HCO_3_ (PDF No. 09-0415).

According to BET measurements, hMnO_2_ has a surface area of 168.4 m^2^/g and an average pore diameter of 6.23 nm (Fig. 2F). The rough outer surface and high surface area of MPCZ NPs are promising for drug delivery. Further experiments were performed using ZPP loaded into the hollow structure of hMnO_2_ NPs. The dose of ZPP loading was evaluated employing a UV–vis spectrometer and HPLC. In order to calculate the amount of ZPP loading, the ZPP that remains in supernatants was subtracted from the amount initially used, and the loading ratios of ZPP was measured to be approximately 4.7% in Fig. 2G and Figure S1. To quantitatively ascertain the content of PDA and NH_4_HCO_3_, MP (MnO_2_-PDA) and MPC (MnO_2_-PDA- NH_4_HCO_3_) NPs were synthesized. Their respective mass fractions of PDA and NH_4_HCO_3_ were 21.3% and 2.1%, which determined by thermogravimetric analysis (TGA) as depicted in Figure S2. Based on the results above, we were able to successfully fabricate MPCZ NPs which contain 71.9% MnO_2_, 21.3% PDA, 2.1% NH_4_HCO_3_, and 4.7% ZPP on a mass basis. As shown in Fig. 2H, Mn 2p XPS spectra were used to further analyze the valence states of Mn. The peaks at binding energies of 653.96 eV and 642.17 eV could be attributed to Mn ^IV^, which are consistent with previous literature data [30, 31]. Besides, a significant change in the zeta potential of MPCZ after coating with PDA can be seen in Fig. 2I, indicating that the nanoparticles were successfully modified at each step. The size distribution of nanoparticles for each step of synthesis by DLS analysis was shown in Figure S3A and S3B. These nanoparticles were larger than in the previous step. All these data confirmed the successful construction of MPCZ NPs.

### In vitro ROS generation and Mn^2+^, ZPP release

**Fig. 3:**
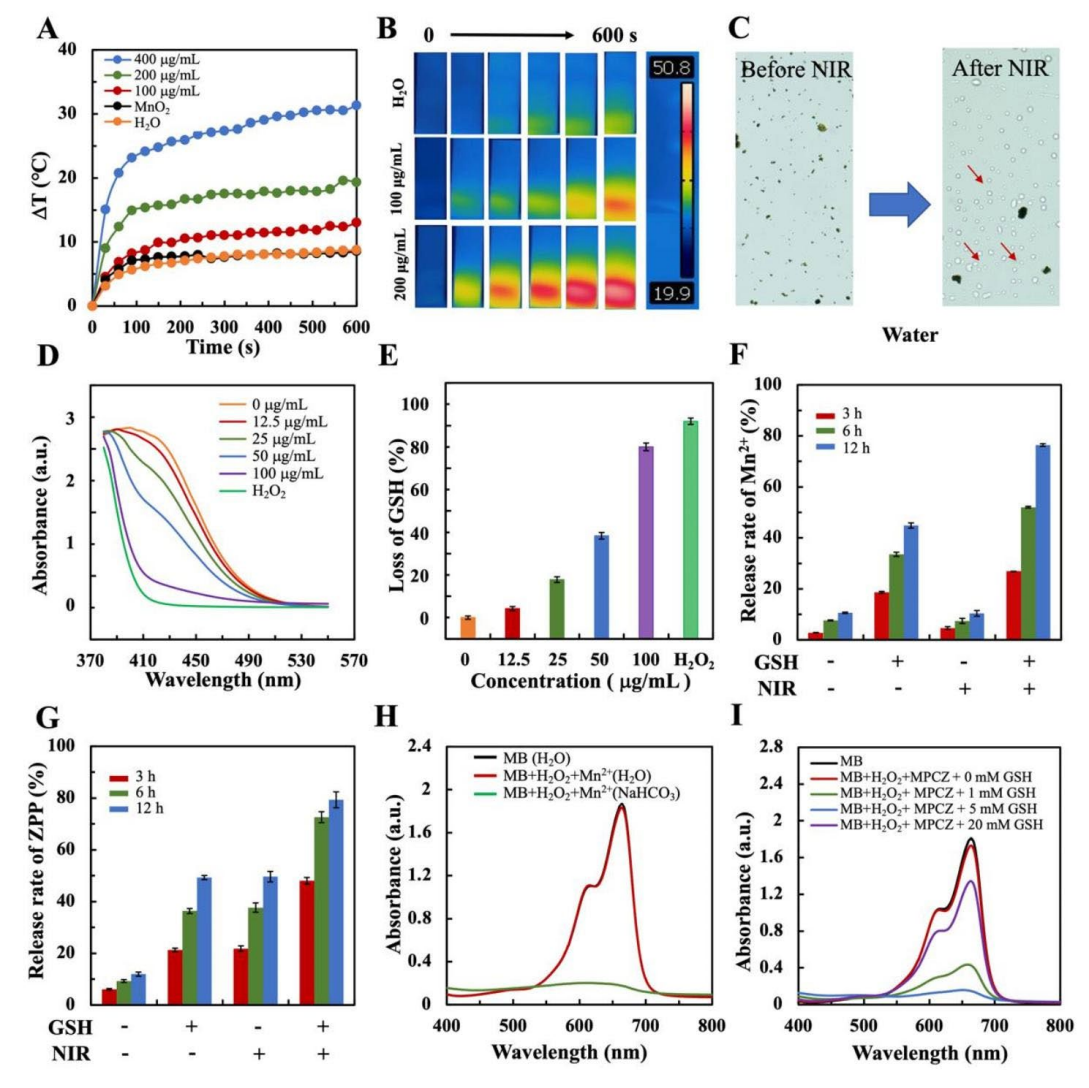
**GSH-dependent MPCZ NPs decomposition and Mn**^**2+**^, **ZPP release behaviors.** Temperature elevation curves (**A**) and infrared thermal images (**B**) of H_2_O and MPCZ (comparable to 100 and 200 µg/mL MPC or 5.0 and 10.0 µg/mL ZPP) suspension under 808 nm laser irradiation (1 W/cm^2^, 10 min). (**C**) Image of MPCZ solution with or without NIR irradiation, the red arrow points to gas bubbles. (**D**) UV − vis spectra of GSH reaction with different concentrations of MPCZ NPs and (**E**) corresponding percent depletion of GSH with NIR irradiation (1 W/cm^2^, 10 min). Percentages of released Mn^2+^ (**F**) and ZPP (**G**) from MPCZ NPs over time with presence or absence of GSH and NIR laser irradiation. UV-vis absorption spectra of MB following Fenton-like reaction-mediated degradation by Mn^2+^ (**H**) and GSH-treated MPCZ NPs (**I**) under 808 nm laser irradiation (1 W/cm^2^, 10 min)

The photothermal properties of MPCZ NPs were evaluated under NIR laser irradiation. The solution temperature of 200 µg/mL MPCZ NPs rose rapidly by 19.3 ºC within 10 min under NIR laser irradiation, suggesting a significant photothermal performances (Fig. 3A). Further investigations demonstrated that the temperature of MPCZ NPs improved with a gradual increase in dose (Fig. 3A), an observation that was reinforced by thermal images taken in the infrared region (Fig. 3B and S4). To attain maximum Fenton reaction efficacy, MPCZ NPs were exposed to NIR laser irradiation for the exploration of gas generation. Lots of bubbles could appear with NIR laser irradiation, but could not or a small number of bubbles appear before laser irradiation in Fig. 3C. As a result of the elevated temperature, the gas produced by NH_4_HCO_3_ disrupted the shell layer of the MPCZ NPs, exposing its internal hMnO_2_ to the tumor microenvironment. As shown in Figure S5, the TEM image of MPCZ NPs after irradiation showed that PDA shell was ruptured, resulting in the surface exposure of MnO_2_ and the release of ZPP. Moreover, the MnO_2_ NPs could degrade rapidly under simulated the tumor microenvironment in Figure S6. As demonstrated in Fig. 3D and E, the consumption of GSH was measured via Ellman’s assay, which indicated that MPCZ NPs had a significant ability to deprive GSH under NIR laser irradiation. Moreover, under NIR laser irradiation, MPCZ NPs were further degraded with the action of high level GSH. Thus, the release of Mn^2+^ and ZPP from MPCZ NPs have also been studied with or not NIR laser irradiation (Fig. 3F and G and S7). In comparison to MPCZ NPs non-NIR laser irradiation, the release speeds of Mn^2+^ and ZPP increased significantly under high level GSH and NIR laser irradiation. Moreover, NIR could cause the rupture of PDA and release of partial ZPP due to decomposition of NH_4_HCO_3_. However, the release of Mn^2+^ requires high GSH and NIR laser irradiation. Thus, the release amounts of ZPP could be higher than that of Mn^2+^ under GSH- and NIR + conditions in Fig. 3F and G. This indicates that the release of Mn^2+^ and ZPP can be accelerated only under the synergic stimulation of exogenous stimulus (NIR laser irradiation) and endogenous stimulus (high level GSH).

Apart from enhancing the cGAS-STING signaling pathway, Mn^2+^ can also trigger the Fenton reaction in a H_2_O_2_-dependent process. By monitoring the oxidative degradation of MB, the production of ·OH could be detected. As shown in Fig. 3H, MB absorbance decreased significantly after treatment with H_2_O_2_ and MnCl_2_ in NaHCO_3_/CO_2_ buffer, but not in aqueous solution after treatment with the same compounds. According to the results, Mn^2+^ and H_2_O_2_ could effectively produce ·OH in physiological conditions. Besides, a further step was to investigate whether MPCZ NPs depleted GSH and enhanced CDT under NIR laser irradiation. The degradation of MB was observed because of MPCZ NPs in the presence of H_2_O_2_ and GSH in Fig. 3I, which can be attributed to the Fenton-like Mn^2+^ release from MPCZ NPs stimulated by GSH and NIR laser irradiation. Moreover, when GSH was excessive (from 5 to 20 mM), this degradation decreased due to its scavenging effect on ·OH. These results indicated that MPCZ NPs are capable of releasing Mn^2+^ and ZPP responsively, as well as efficiently generating abundant ROS.

### Intracellular anti-tumor effect and cGAS-STING activation of MPCZ NPs

**Fig. 4:**
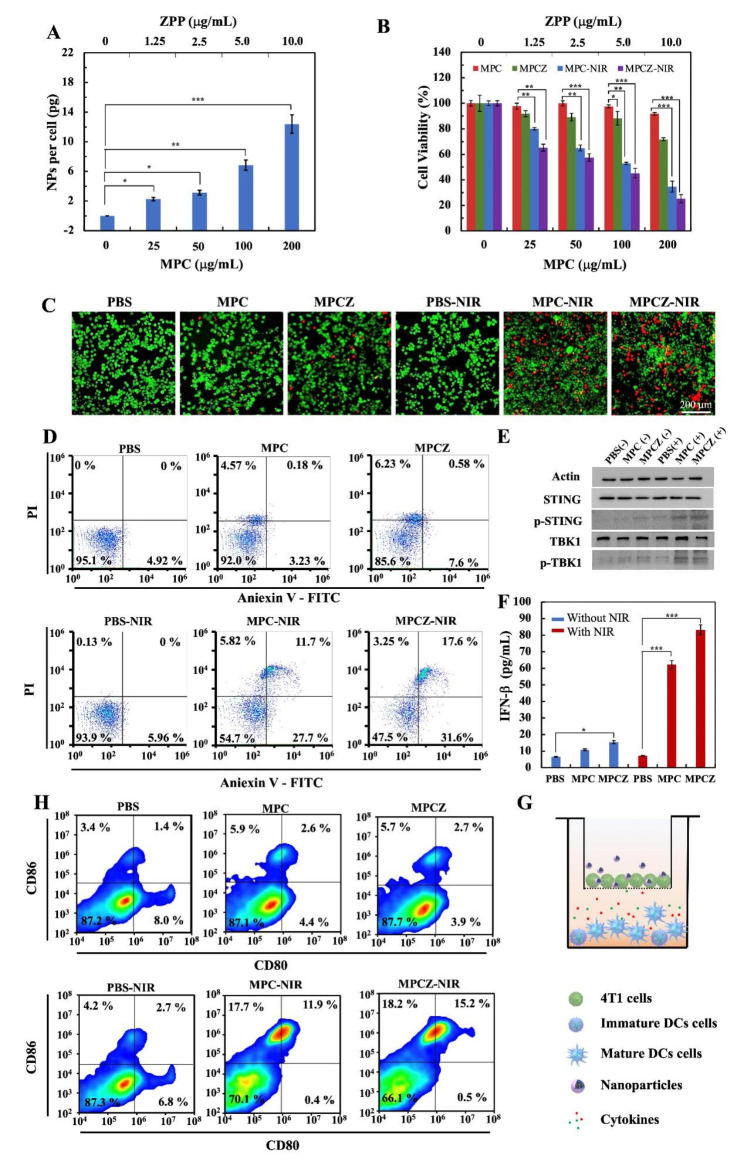
**Intracellular toxicity evaluation and amplification effects of cGAS-STING signaling pathway.** (**A**) The cellular uptake of MPCZ NPs at different concentrations was analyzed by ICP-MS. The labeled asterisk represents statistical significance compared with control group. * *p* < 0.05, ** *p <* 0.01, *** *p <* 0.001. (**B**) – (**D**) Cell viability analysis of 4T1 cells treated by PBS, MPC, MPCZ NPs (comparable to 25, 50, 100 and 200 µg/mL MPC or 1.25, 2.5, 5.0 and 10.0 µg/mL ZPP) (n = 3). MTT (**B**), Live/dead staining (**C**) and Fluorescein-annexin V and PI staining assays (**D**) of 4T1 cells treated by PBS, MPC, MPCZ NPs (comparable to 100 µg/mL MPC or 5.0 µg/mL ZPP) for 6 h, then irradiated with or without 808 nm laser (1 W/cm^2^) for 10 min and incubated for following 18 h. Labeled asterisk represents statistical significance compared with MPC NPs without 808 nm laser irradiation via one-way ANOVA with the Tukey post-hoc test. * *p* < 0.05, ** *p <* 0.01, *** *p <* 0.001. (**E**) The protein expression levels of STING, p-STING, p-TBK1 and TBK1 were analyzed by western blot in 4T1 cells. (**F**) The release of INF-β from PBS, MPC NPs and MPCZ NPs with or without 808 nm laser irradiation detected by ELISA kit (n = 3). For (**E**) and (**F**), cells were treated by PBS, MPC NPs and MPCZ NPs (comparable to 25 µg/mL MPC or 1.25 µg/mL ZPP) for 6 h and irradiated with or without 808 nm laser (1 W/cm^2^) for 10 min for following 6 h incubation. Based on one-way ANOVA with Tukey post-hoc analysis, the asterisks represent statistical significance in comparison to the PBS group. *p < 0.05, ***p < 0.001. (**G**) Schematic illustration of the 4T1/DCs transwell system treated with different NPs. (**H**) Flow cytometry measurements of DCs surface for CD86 and CD80 expression after different treatments

The cellular uptake capacity of MPCZ NPs was studied prior to evaluating the anti-tumor effect in vivo. 4T1 cells incubated with different concentrations of MPCZNPs were digested  and analyzed by ICP-MS. The intracellular MPCZ NPs level increased in a dose-dependent manner, indicating significant uptake of MPCZ NPs in Fig. 4A. To explore the antitumor activity of MPCZ NPs at the cellular level, MTT assay was adopted to investigate cell viability. It could be seen that the cell viability of 4T1 cells treated with MPC NPs and MPCZ NPs was weakly reduced with concentrations without NIR laser irradiation, while MPC NPs and MPCZ NPs showed significant toxicity at 100 µg/mL and 200 µg/mL under NIR laser irradiation in Fig. 4B. The results showed that NIR laser irradiation was an important exogenous stimulus to turn on the toxicity of MPC NPs and MPCZ NPs. Moreover, the results similarly to those of MTT were also obtained by the cell live/dead staining assay in Fig. 4C. According to Fig. 4D, fluorescein-annexin V and PI staining assays were used to investigate the association between MPCZ NP toxicity and apoptosis. Under NIR laser irradiation, the induction rates of PBS, MPC NPs and MPCZ NPs on early apoptotic cells were 5.96%, 27.7% and 31.6%, respectively, and the induction rates of late apoptotic cells were 0, 11.7% and 17.6%, respectively, indicating that MPCZ NPs had a stronger ability to induce apoptosis. In contrast, MPC NPs and MPCZ NPs induced a small number of apoptotic cells without NIR laser irradiation, which was consistent with the results of MTT and the live/dead staining assay. This indicated that the cytotoxicity of MPCZ NPs is mainly related to apoptosis.

Considering the generation of more ROS and destruction of mitochondria in tumor cells, it will force the production of cytoplasmic dsDNA, thus activating cGAS-STING signaling pathway. In order to explore the activation effect of MPCZ NPs on STING signaling pathway, the downstream relative indicators STING, phosphorylated STING (p-STING), TBK1 and phosphorylated TBK1 (p-TBK1) were further analyzed by western blot analysis. As illustrated in Fig. 4E, the expression of p-STING and p-TBK1 in MPC NPs and MPCZ NPs groups was significantly higher than that in other groups under NIR laser irradiation. In addition, to further explore the role of MPCZ NPs on STING signal transduction, Elisa assay was used to evaluate the release of INF-β and CXCL10. As shown in Fig. 4F and Figure S8, MPCZ NPs significantly enhanced the transcription of INF-β and CXCL10 in 4T1 cells under NIR laser irradiation. Furthermore, the production of IFNs could enhance tumor-specific antigen presentation on DCs. To assess the maturation of DCs, 4T1 cells treated by PBS, MPC, MPCZ NPs were plated into upper compartment of transwell system and DCs were plated into the bottom of the transwell system in Fig. 4G. As shown in Fig. 4H, under NIR laser irradiation, the MPC NPs and MPCZ NPs groups stimulated almost 11.9% and 15.2% DCs maturation, compared to 2.7% of PBS group and non-NIR laser irradiation groups (1.4%, 2.6%, 2.7%). These results suggest that CDT combined with Mn^2+^ release can amplify STING signaling to promote efficiently DCs maturation and improve intracellular anti-tumor immunotherapy effect.

### Intracellular enhanced ROS production with an “open source” and “reduce expenditure” strategy

**Fig. 5:**
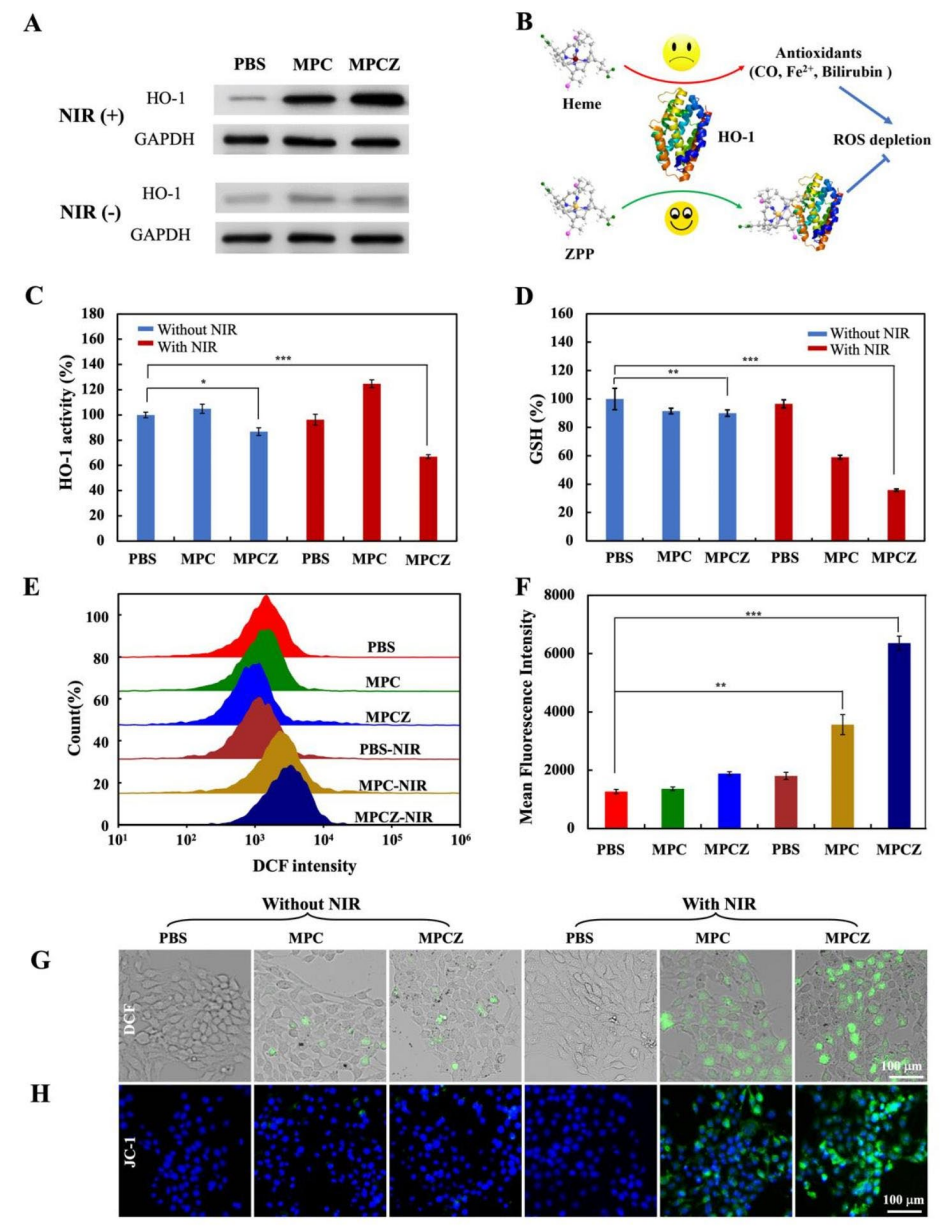
**Intracellular oxidative stress responses.** (**A**) The western blotting analysis of HO-1 expression in PBS, MPC and MPCZ groups with or without 808 nm laser irradiation. (**B**) Schematic illustration of ZPP interacting with HO-1 by competitively with heme to inhibit the antioxidant activity of tumor cells. (**C**) HO-1 activity by bilirubin assay. (**D**) Intracellular GSH levels. (**E**) and (**F**) Flow cytometry analysis of ROS in 4T1 cells treated with different nanoparticles. (**G**) DCF fluorescence imaging of MPCZ NPs in 4T1 cells induced by ROS. (**H**) Fluorescence images of 4T1 cells stained with JC-1 (green) were used to analyze the depolarization of mitochondrial membrane. For (**A**) and (**C**) to (**H**), 4T1 cells were treated with MPC and MPCZ NPs (comparable to 25 µg/mL MPC or 1.25 µg/mL ZPP) for 6 h, then irradiated with or without 808 nm laser (1 W/cm^2^) for 10 min and incubated for following 6 h. For (**C**), (**D**) and (**F**), the asterisks represent statistical significance in comparison to the PBS group based on one-way ANOVA with Tukey post-hoc analysis. *p < 0.05, ** *p <* 0.01, ***p < 0.001

Based on the excellent ROS generation performance of MPCZ NPs, the effects of adopting open source and reduce expenditure strategy at the cellular level were further explored. To clarify the effect of “open source”, 4T1 cells were cultured with PBS, MPC and MPCZ NPs for 12 h with or without NIR laser irradiation and western blot analysis was performed to investigate the expression of HO-1 in cells. The result indicated that under NIR laser irradiation, MPC NPs and MPCZ NPs groups upregulated HO-1 expression more significantly than various groups (PBS, MPC NPs, MPCZ NPs) without NIR laser irradiation as shown in Fig. 5A. This shows that MPC NPs and MPCZ NPs may enhance oxidative stress response of tumor cells under NIR laser irradiation.

In terms of “reduce expenditure”, the responsive release of ZPP and the consumption of GSH could decrease the antioxidant activity of tumor cells. Recent work has reported that HO-1 plays a significant role in antioxidant defense in tumor cells [32]. As a result of ROS attacking cells and disrupting redox homeostasis, HO-1 can catalyze the heme molecules into antioxidants such as carbon monoxide (CO), ferrous iron (Fe^2+^), and biliverdin. These antioxidants would contribute to ROS depletion. As illustrated in Fig. 5B, the responsive release of ZPP in tumor cells competitively inhibits the binding of HO-1 to heme, thereby inhibiting the activity of HO-1 and blocking the antioxidant capacity of tumor cells. MPCZ NPs were investigated for their effect on activity of HO-1 by the following methods, bilirubin assay was assessed by PBS, MPC NPs, MPCZ NPs treated 4T1 cells with or without NIR laser irradiation in Fig. 5C. The result shows that the HO-1 activity of MPCZ NPs groups with and without NIR laser irradiation decreased than PBS and MPC NPs group, which indicateds ZPP has a strong inhibition effect on HO-1 activity. Moreover, the HO-1 activity of the MPC NPs under NIR laser irradiation was higher than the control group in Fig. 5 C. This is attributed to the antioxidant defenses of tumor cells against external stimuli. HO-1 overexpression and consequent HO-1 activity increase is responsible for Tier 1 level of antioxidant defense based on hierarchical oxidative stress toxicological paradigm. Subsequently, to investigate the consumption of GSH, DTNB assay was used to measure the GSH content of 4T1 cells incubated by PBS, MPZ NPs and MPCZ NPs with or without NIR laser irradiation in Fig. 5D. The results imply that MP NPs and MPCZ NPs groups significantly reduced the GSH content under NIR laser irradiation, indicating that it had a strong consumption capacity of GSH.

Due to the open source and reduced expenditure strategy of MPCZ NPs, the DCFH-DA probe was also employed to assess the level of intracellular oxidative stress. As shown in Fig. 5E and 5F, MPC and MPCZ NPs under NIR laser irradiation induced higher intracellular ROS generation than other groups by flow cytometry analysis. In addition, when DCFH-DA is exposed to oxidative stress, it converts into green fluorescent DCF. The 4T1 cells treated by MPCZ NPs showed stronger green fluorescence under NIR laser irradiation than other groups by fluorescence imaging, attributing to intense oxidative stress in the cells in Fig. 5G. The release of mitochondrial DNA can result from excessive ROS damage to mitochondria. Fluorescence probes like JC-1 are widely employed to detect the degree of depolarization in mitochondrial membrane potentials, reflected by green fluorescence intensity ratios in Fig. 5H. Above data indicated that MPCZ NPs has excellent ROS generation ability under NIR laser irradiation, which can trigger intense oxidative stress responses and mitochondrial destruction.

### In vivo the anti-tumor and immunotherapy effects of MPCZ NPs

**Fig. 6:**
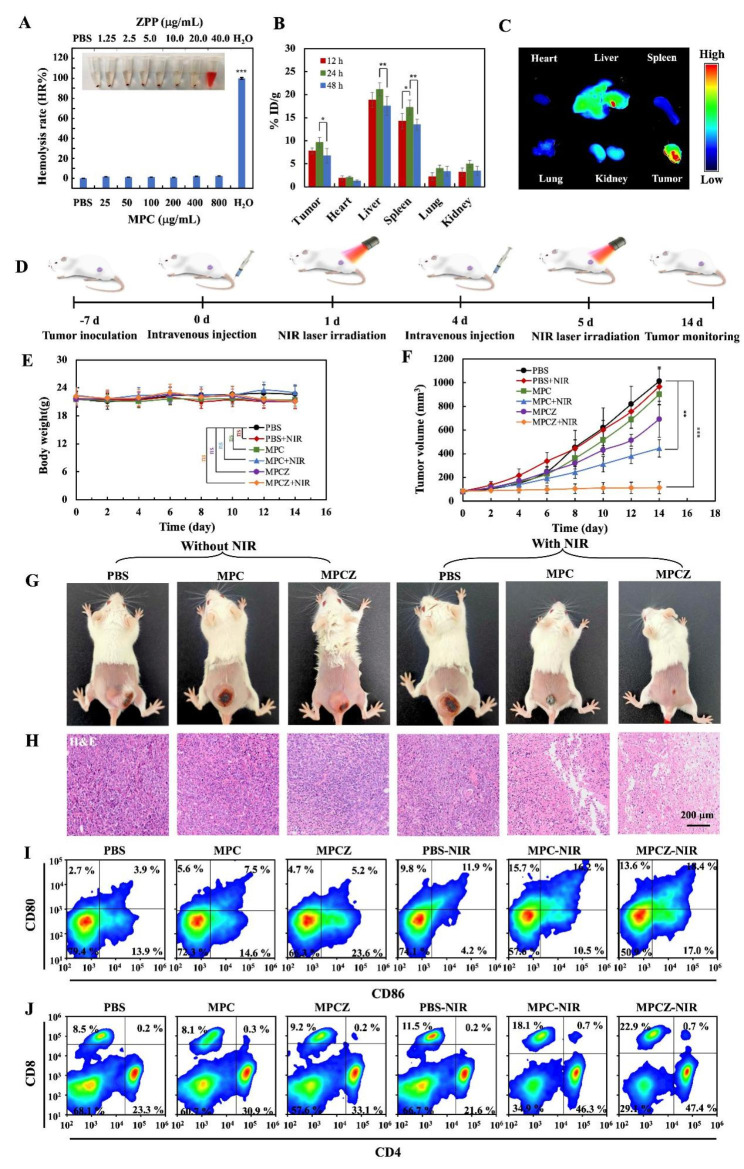
**In vivo the anti-tumor and immunotherapy effects of MPCZ NPs.** (**A**) Digital photograph of hemolysis test and hemolysis rate (HR%) of MPCZ NPs. (**B**) ICP-MS analysis of Mn elements in tissues dissected from mice at 12, 24 or 48 h post-administration with MPCZ NPs (data expressed as percentage of the injected dose per gram of tissue (% ID/g)). (**C**) Fluorescence images of main organs and tumors at 24 h post-injection of MPCZ NPs. (**D**) Schematic illustration of MPCZ NPs therapy. (**E**) Weight change of mice during the treatment period. (**F**) Tumor growth curves of PBS, MPC NPs and MPCZ NPs (comparable to 20 mg/kg mouse for MPC and 1 mg/kg mouse for ZPP) treated 4T1 tumor-bearing mice with or without 808 nm laser irradiation. (**G**) Images of tumor-bearing mice after 14 days of different treatments. (H) Tumor tissue staining by H&E after 14 days of treatments. (**I**) Frequency of CD80^+^ CD86^+^ DCs in tumor-draining lymph nodes (gate on CD11c^+^ DCs). (**J**) Frequency of CD4^+^ and CD8^+^ DCs in tumor-draining lymph nodes (gate on CD3^+^ T cells). For (**A**), (**B**), (**E**) and (**F**), the asterisks represent statistical significance in comparison to the PBS group based on one-way ANOVA with Tukey post-hoc analysis. *p < 0.05, ** *p <* 0.01, ***p < 0.001

The amplification effect of MPCZ NPs on the cGAS-STING signaling pathway in vitro has prompted us to evaluate its antitumor immunotherapeutic potential in vivo. First, nanomedicine can be further used in cancer treatment only if it has low toxic effects on the body. To investigate its toxicity, hemolysis assay, blood type data analysis and H&E staining of the heart, liver, spleen, lung and kidney were evaluated. For the hemolysis rate of MPCZ NPs in Fig. 6A, the hemolysis rates of MPCZ NPs at different concentrations were all less than 10%, indicating that MPCZ NPs is safe to be used in injection. Then the biodistribution of MPCZ NPs at 12 h, 24 and 48 h after intravenous injection was assessed by ICP-MS. In Fig. 6B, we can see that MPCZ NPs could accumulate significantly in tumor tissues, with the most effective accumulation occurring 24 h after injection. Cy5-loaded MPCZ NPs also showed significant tumor accumulation behavior at 24 h in contrast to the free Cy5 group in Figure 6 C and S9.

Subsequently, through the evaluation of the blood data of mice injected with PBS or MPCZ NPs (with and without NIR), the liver function (Figure S10), kidney function (Figure S11), blood biochemistry and hematology analysis (Figure S12) of mice showed no obvious toxicity. Moreover, in order to explore the toxic side effects of MPCZ NPs injection on various organs, H&E staining was used to evaluate. As depicted in Figure S13 and S14, H&E staining of major organs further demonstrated that MPCZ NPs had good histocompatibility. These data further support that the good biocompatibility and the tumor accumulation of MPCZ NPs in vivo.

In addition, to further explore the anti-tumor effect of MPCZ NPs in vivo, 4T1 tumor-bearing mice were treated once every three days and twice during the treatment period in Fig. 6D. During the treatment period, MPCZ NPs injection had no significant effect on the body weight of mice in Fig. 6E. After treatment, MPCZ NPs group showed the most significant tumor inhibitory effect under NIR laser irradiation, which was consistent with the in vitro antitumor effect compared with PBS and MPC NPs groups and non-laser irradiation groups (Fig. 6F, 6G and S15). Moreover, the abscopal tumors were suppressed for the mice treated with MPCZ NPs under NIR laser irradiation in the abscopal tumors model, which was due to the anti-tumor immune response in Figure S16. To further clarify the therapeutic effect of MPCZ NPs, H&E staining was used for histological analysis of tumors. By evaluating the pathological changes of tumor sections at the end of treatment, the MPCZ NPs group caused severe cell damage and inflammatory lesions under NIR laser irradiation, while the remaining group caused minor damage or no significant histological changes in Fig. 6H. The TUNEL experiment further confirmed that MPCZ NPs significantly enhanced the apoptosis of cancer cells under NIR laser irradiation in Figure S17. To better elucidate the role of immunotherapy in antitumor process, maturation of DCs in tumor draining lymph nodes was analyzed. As shown in Fig. 6I, the ratios of DCs maturation in the MPC NPs (16.2%) and MPCZ NPs (18.4%) groups under NIR laser irradiation were significantly higher than that in the PBS group and non-NIR laser irradiation groups (3.9%, 7.5%, 5.2%) in tumor draining lymph nodes. Moreover, the frequency of CD4^+^ or CD8^+^ T lymphocytes in the tumor-draining lymph node was further investigated by flow cytometry. The infiltration of CD8^+^ T lymphocytes of mice with PBS + NIR, MPC NPs + NIR and MPCZ NPs + NIR treatment were 11.5%, 18.1% and 22.9%, and the CD4^+^ T lymphocytes were 21.6%, 46.3% and 47.4%, respectively. However, all of the treated mice without NIR laser irradiation retained low-frequency CD4^+^ T lymphocytes and CD8^+^ T cells in Fig. 6J. In addition, the infiltrations of CD4^+^ and CD8^+^ T lymphocytes of mice of MPC NPs + NIR and MPCZ NPs + NIR groups were significantly higher than other groups in spleen (Figure S18). Furthermore, under NIR laser irradiation, CD4^+^ and CD8^+^ T cells in tumors were increased after MPCZ NPs treatment (Figure S19), confirming that MPCZ NPs treatment can activate the anti-tumor immune response based on macrophages and T cells. Therefore, activation of cGAS-STING signal can promote the infiltration of CD8^+^ and CD4^+^ T cells at the tumor site and the cross-presentation of DCs, thus improving the effect of immunotherapy on tumors.

## Discussion

Harnessing the immune system to attack cancer is a major goal of immunotherapy [33]. Immunotherapy can be divided into innate immunity and adaptive immunity according to the specific level of antigen. The antigen receptors of the adaptive immune response are often involved in determining lymphocyte function [34]. Adaptive immunity in which T antigen receptors are the mainstay includes immune checkpoint blockade, adoptive cellular therapies, and cancer vaccines. They play an important role in “unleashing” powerful T cell responses, injecting anti-tumor immune cells into the body, and designing preventive or therapeutic activities, respectively [35]. However, adaptive immunity dominated by cancer antigen-specific T cells do not work in isolation. Instead, their activity is largely determined by cellular signals and innate immune mechanisms [36]. Therefore, the surveillance of tumor immunity depends on the innate immune system, so the optimal connection between innate and adaptive immunity is essential for tumor therapy. Although various damage-related molecular patterns can play a certain alarm role in innate immunity, abnormal DNA activation through the cGAS-STING signaling pathway is particularly important for the endogenous perception of transformed or transformed cells [36]. cGAS-STING signaling pathway has become a key innate immune pathway, promoting different immune responses and affecting tumor progression and metastasis [37]. Downstream cytokines regulated by cGAS-STING, especially IFNs type I, are the bridge between innate immunity and adaptive immunity [38]. In addition, STING activation can also induce autophagy and cell death responses independent of induction by downstream transcriptional programs [39].

The activation of cGAS-STING signaling pathway originates from the connection of cGAS to abnormal DNA in the cytoplasm. Furthermore, ATP and GTP are catalyzed by cGAS to generate cGAMP, thus activating the cGAS-STING signaling pathway. In tumor cells, abnormal DNA accumulation is due to chromosomal instability, replication pressure, reactivation of endogenous reverse transcription factors or release of mitochondrial DNA or defects in DNA damage response. These DNA can stimulate the cGAS-cGAMP-STING signaling pathway and up-regulate type I IFNs pro-inflammatory factors and chemokines by activating IRF3 and NF-kB transcription factors. An effective way to further promote tumor cell accumulation of abnormal DNA is by releasing mitochondrial DNA. There is increasing evidence that mitochondria are a major source of intracellular ROS (~ 90% of ROS) [40]. Furthermore, excessive production of cellular ROS is associated with mitochondrial dysfunction, characterized by loss of mitochondrial membrane potential (ΔΨm), increased mitochondrial mass and increased mitochondrial DNA (mtDNA) fragmentation. Excess ROS alters the mitochondrial genome and increases protein oxidation, thereby disrupting the antioxidant defenses of the cellular compartment and leading to mitochondrial damage. It is possible to accumulate excessive ROS in tumor cells by taking advantage of high hydrogen peroxide levels and conditions of GSH, and then use the injured mitochondria to release dsDNA [41, 42]. Adopting open source and reduce expenditure strategy is an efficient way to amplify ROS generation. Multi-pathway reduction of ROS consumption and increase of ROS production are the main means, which have demonstrated superior anti-tumor effects in vitro and in vivo [25, 43–45].

Metal immunotherapy is the use of metal ions to regulate immune function, and then regulate immunotherapy. For example, calcium ion (Ca^2+^) can be attached to the phosphate group of negative phospholipids, thereby neutralizing the negative charge of phospholipids and regulating the activation of T cells [46]. Magnesium ion (Mg^2+^) increased the expression of NKG_2_D and restored the natural cytotoxicity killer cells (NK) and T cells [47]. Besides, zinc ion (Zn^2+^) and Mn^2+^ can active cGAS-STING signaling pathway [48]. Mn^2+^ improves the sensitivity of cGAS to intracellular DNA and its catalytic activity, which can promote cGAS to generate cGAMP under the stimulation of low concentration of DNA [49]. In addition, Mn^2+^ activated cGAS undergoes a similar conformational change to DNA-activated cGAS, but forms a unique helix to expand the catalytic pocket, allowing substrate entry and cGAMP synthesis [50, 51]. Furthermore, Mn^2+^ promotes the dimerization of c-di-GMP and 3’ 3’-cGAMP, increasing enhanced cGAMP-STING affinity [52].

## Conclusions

In summary, we constructed MPCZ NPs to activate responsively and efficiently the cGAS-STING signaling pathway by loading PDA embedded with NH_4_HCO_3_ and coating ZPP outside hMnO_2_ NPs. Under the action of exogenous NIR laser irradiation and endogenous GSH of high level, MPCZ NPs can synergically regulate the release of Mn^2+^ in tumor cells and generate more ROS by open source and reduce expenditure strategy. The responsive release or production of Mn^2+^ and ROS effectively enhance STING signaling pathway and amplify innate immunity. The MPCZ NPs demonstrated effective tumor growth inhibition in 4T1 tumor-bearing mice model, providing potential for clinical application in promoting antitumor immunotherapy.

## Electronic supplementary material

Below is the link to the electronic supplementary material.

Supplementary Material 1

## Data Availability

The data and analysis generated in this study are included in the manuscript and Supporting Information file.

## List of Abbreviations

cGAS-STING: The cyclic guanosine monophosphate-adenosine monophosphate synthase-stimulator of interferon genes
MPCZ NPs: Manganese-based nanoplatform
Mn^2+^: Manganese ions
ROS: Reactive oxygen species
MnO_2_: Manganese dioxide
ZPP: Zinc protoporphyrin IX
PDA: Polydopamine
MTT: Methyl thiazolyl tetrazolium
·OH: Hydroxyl radical
CAR-T therapy: Chimeric antigen receptor T-cell immunotherapy
ICB: Immune checkpoint blockade
GMP-AMP: The cyclic guanosine monophosphate-adenosine monophosphate
cGAS: GMP-AMP synthase
ATP: Adenosine triphosphate
GTP: Guanosine triphosphate
IFNs: Type I interferons
DCs: Dendritic cells
CDN: Cyclic dinucleotide
dsDNA: Double-stranded DNA
EPR: Enhanced permeability and retention
CDT: Chemodynamic therapy
PTT: Photothermal therapy
HO-1: Heme oxygenase
TEM: Transmission electron microscopy
SEM: Scanning Electron Microscope
FTIR: Fourier Transform Infrared Spectroscopy
BET: Brunauer-Emmett-Teller
DLS: Dynamic Light Scattering
FLIR: Infrared Thermal Imager
ICP-MS: Inductively coupled plasma mass spectrometry
CO: Carbon monoxide
Fe^2+^: Ferrous iron
p-STING: Phosphorylated STING
p-TBK1: TBK1 and phosphorylated TBK1
ΔΨm: Mitochondrial membrane potential
mtDNA: Mitochondrial DNA
Ca^2+^: Calcium ion
Mg^2+^: Magnesium ion
Zn^2+^: Zinc ion
NK: Natural cytotoxicity killer cells

## References

[1] McNutt M (2013). Cancer Immunotherapy Science.

[2] Huang AC, Postow MA, Orlowski RJ, Mick R, Bengsch B, Manne S (2017). T-cell invigoration to tumour burden ratio associated with anti-PD-1 response. Nature.

[3] Ni K, Luo T, Culbert A, Kaufmann M, Jiang X, Lin W (2020). Nanoscale Metal-Organic Framework Co-delivers TLR-7 agonists and Anti-CD47 antibodies to modulate Macrophages and Orchestrate Cancer Immunotherapy. J Am Chem Soc.

[4] Jiang H, Guo Y, Wei C, Hu P, Shi J (2021). Nanocatalytic innate immunity activation by mitochondrial DNA oxidative damage for Tumor-Specific Therapy. Adv Mater.

[5] Gou S, Liu W, Wang S, Chen G, Chen Z, Qiu L (2021). Engineered Nanovaccine Targeting Clec9a(+) dendritic cells remarkably enhances the Cancer Immunotherapy Effects of STING Agonist. Nano Lett.

[6] Rameshbabu S, Labadie BW, Argulian A, Patnaik A (2021). Targeting Innate Immunity in Cancer Therapy. Vaccines (Basel).

[7] Du B, Bai Y, Jiao Q, Zhao M, Pang M, Ma H (2022). Simultaneous innate immunity activation and immunosuppression improvement by biodegradable nanoplatform for boosting antitumor chemo-immunotherapy. Chem Eng J.

[8] Song W, Song SJ, Kuang J, Yang H, Yu T, Yang F (2022). Activating Innate immunity by a STING Signal Amplifier for local and systemic immunotherapy. ACS Nano.

[9] Zhang X, Bai XC, Chen ZJ (2020). Structures and mechanisms in the cGAS-STING innate immunity pathway. Immunity.

[10] Li Z, Chu Z, Yang J, Qian H, Xu J, Chen B (2022). Immunogenic cell death augmented by Manganese Zinc Sulfide Nanoparticles for metastatic Melanoma Immunotherapy. ACS Nano.

[11] Guo W, Chen Z, Li Z, Huang H, Ren Y, Zhao B (2022). Improved immunotherapy for gastric cancer by nanocomposites with capability of triggering dual-damage of Nuclear/Mitochondrial DNA and cGAS/STING-Mediated innate immunity. Chem Eng J.

[12] Michalski S, de Oliveira Mann CC, Stafford CA, Witte G, Bartho J, Lammens K (2020). Structural basis for sequestration and autoinhibition of cGAS by chromatin. Nature.

[13] Chen Q, Sun L, Chen ZJ (2016). Regulation and function of the cGAS-STING pathway of cytosolic DNA sensing. Nat Immunol.

[14] Shi J, Liu C, Luo S, Cao T, Lin B, Zhou M (2021). STING agonist and IDO inhibitor combination therapy inhibits tumor progression in murine models of colorectal cancer. Cell Immunol.

[15] Woo S-R, Fuertes Mercedes B, Corrales L, Spranger S, Furdyna Michael J, Leung Michael YK (2014). STING-Dependent cytosolic DNA sensing mediates Innate Immune Recognition of Immunogenic Tumors. Immunity.

[16] Guo H, Huang J, Tan Y, Wu W, Huang T, Zhang N (2022). Nanodrug shows spatiotemporally controlled release of anti-PD-L1 antibody and STING agonist to effectively inhibit tumor progression after radiofrequency ablation. Nano Today.

[17] Harrington KJ, Brody J, Ingham M, Strauss J, Cemerski S, Wang M (2018). Preliminary results of the first-in-human (FIH) study of MK-1454, an agonist of stimulator of interferon genes (STING), as monotherapy or in combination with pembrolizumab (pembro) in patients with advanced solid tumors or lymphomas. Ann Oncol.

[18] Junkins RD, Gallovic MD, Johnson BM, Collier MA, Watkins-Schulz R, Cheng N (2018). A robust microparticle platform for a STING-targeted adjuvant that enhances both humoral and cellular immunity during vaccination. J Control Release.

[19] Baird JR, Friedman D, Cottam B, Dubensky TW, Kanne DB, Bambina S (2016). Radiotherapy Combined with Novel STING-Targeting oligonucleotides results in regression of established tumors. Cancer Res.

[20] Cheng Z, Li M, Dey R, Chen Y (2021). Nanomaterials for cancer therapy: current progress and perspectives. J Hematol Oncol.

[21] Sundaram A, Peng L, Chai L, Xie Z, Ponraj JS, Wang X (2020). Advanced nanomaterials for hypoxia tumor therapy: challenges and solutions. Nanoscale.

[22] Quader S, Kataoka K (2017). Nanomaterial-enabled Cancer Therapy. Mol Ther.

[23] Liu C, Liu B, Zhao J, Di Z, Chen D, Gu Z (2020). Nd(3+) -Sensitized Upconversion Metal-Organic Frameworks for Mitochondria-Targeted amplified photodynamic therapy. Angew Chem Int Ed.

[24] Zhou S, Xu J, Dai Y, Wei Y, Chen L, Feng W (2022). Engineering tumor-specific catalytic nanosystem for NIR-II photothermal-augmented and synergistic starvation/chemodynamic nanotherapy. Biomater Res.

[25] Liu C, Cao Y, Cheng Y, Wang D, Xu T, Su L (2020). An open source and reduce expenditure ROS generation strategy for chemodynamic/photodynamic synergistic therapy. Nat Commun.

[26] Hou L, Tian C, Yan Y, Zhang L, Zhang H, Zhang Z (2020). Manganese-based Nanoactivator Optimizes Cancer Immunotherapy via enhancing Innate Immunity. ACS Nano.

[27] Zheng R, Cheng Y, Qi F, Wu Y, Han X, Yan J (2021). Biodegradable copper-based nanoparticles augmented chemodynamic therapy through deep penetration and suppressing antioxidant activity in tumors. Adv Healthc Mater.

[28] Sang Y, Cao F, Li W, Zhang L, You Y, Deng Q (2020). Bioinspired Construction of a Nanozyme-Based H2O2 homeostasis disruptor for intensive chemodynamic therapy. J Am Chem Soc.

[29] Greene A, Hashemi J, Kang Y (2021). Development of MnO2 hollow nanoparticles for potential drug delivery applications. Nanotechnology.

[30] Lu Q, Chen R, Du S, Chen C, Pan Y, Luan X (2022). Activation of the cGAS-STING pathway combined with CRISPR-Cas9 gene editing triggering long-term immunotherapy. Biomaterials.

[31] Huang Y, Wu S, Zhang L, Deng Q, Ren J, Qu X (2022). A metabolic multistage glutathione depletion used for Tumor-Specific Chemodynamic Therapy. ACS Nano.

[32] Cheng Y, Chang Y, Feng Y, Jian H, Wu X, Zheng R (2019). Bismuth Sulfide Nanorods with Retractable Zinc Protoporphyrin Molecules for suppressing innate antioxidant Defense System and strengthening Phototherapeutic Effects. Adv Mater.

[33] Waldmann TA. Immunotherapy: past, present and future.Nat Med. 2003;9. 10.1038/nm0303-269 12612576

[34] Vivier E, Malissen B (2005). Innate and adaptive immunity: specificities and signaling hierarchies revisited. Nat Immunol.

[35] Waldman AD, Fritz JM, Lenardo MJ (2020). A guide to cancer immunotherapy: from T cell basic science to clinical practice. Nat Rev Immunol.

[36] Burdette DL, Monroe KM, Sotelo-Troha K, Iwig JS, Eckert B, Hyodo M (2011). STING is a direct innate immune sensor of cyclic di-GMP. Nature.

[37] Ishikawa H, Ma Z, Barber GN (2009). STING regulates intracellular DNA-mediated, type I interferon-dependent innate immunity. Nature.

[38] Zheng J, Mo J, Zhu T, Zhuo W, Yi Y, Hu S (2020). Comprehensive elaboration of the cGAS-STING signaling axis in cancer development and immunotherapy. Mol Cancer.

[39] Samson N, Ablasser A (2022). The cGAS-STING pathway and cancer. Nat Cancer.

[40] Diebold L, Chandel NS (2016). Mitochondrial ROS regulation of proliferating cells. Free Radicals Biol Med.

[41] Zhao M, Wang Y, Li L, Liu S, Wang C, Yuan Y (2021). Mitochondrial ROS promote mitochondrial dysfunction and inflammation in ischemic acute kidney injury by disrupting TFAM-mediated mtDNA maintenance. Theranostics.

[42] Rizwan H, Pal S, Sabnam S, Pal A (2020). High glucose augments ROS generation regulates mitochondrial dysfunction and apoptosis via stress signalling cascades in keratinocytes. Life Sci.

[43] Xu X, Huang B, Zeng Z, Chen J, Huang Z, Guan Z (2020). Broaden sources and reduce expenditure: Tumor-specific transformable oxidative stress nanoamplifier enabling economized photodynamic therapy for reinforced oxidation therapy. Theranostics.

[44] Chen M, Zhao S, Zhu J, Feng E, Lv F, Chen W (2022). Open-source and reduced-expenditure nanosystem with ROS self-amplification and glutathione depletion for simultaneous augmented Chemodynamic/Photodynamic therapy. ACS Appl Mater Interfaces.

[45] Zhang H, Cao F, Zhu L, Hou L, Zhang Z (2020). An Ultrasound-Triggered ROS sustained supplier based on Open Source and reduce expenditure strategy for Colon cancer therapy. ChemNanoMat.

[46] Shi X, Bi Y, Yang W, Guo X, Jiang Y, Wan C (2013). Ca2 + regulates T-cell receptor activation by modulating the charge property of lipids. Nature.

[47] Chaigne-Delalande B, Li FY, O’Connor GM, Lukacs MJ, Jiang P, Zheng L (2013). Mg2 + regulates cytotoxic functions of NK and CD8 T cells in chronic EBV infection through NKG2D. Science.

[48] Sun X, Zhang Y, Li J, Park KS, Han K, Zhou X (2021). Amplifying STING activation by cyclic dinucleotide–manganese particles for local and systemic cancer metalloimmunotherapy. Nat Nanotechnol.

[49] Wang C, Guan Y, Lv M, Zhang R, Guo Z, Wei X (2018). Manganese increases the sensitivity of the cGAS-STING pathway for double-stranded DNA and is required for the host defense against DNA viruses. Immunity.

[50] Yu X, Zhao Z, Jiang Z (2022). Recent progress on the activation of the cGAS-STING pathway and its regulation by biomolecular condensation. J Mol Cell Biol.

[51] Zhao Z, Ma Z, Wang B, Guan Y, Su XD, Jiang Z (2020). Mn(2+) directly activates cGAS and structural analysis suggests Mn(2+) induces a noncanonical Catalytic synthesis of 2’3’-cGAMP. Cell Rep.

[52] Roembke BT, Zhou J, Zheng Y, Sayre D, Lizardo A, Bernard L (2014). A cyclic dinucleotide containing 2-aminopurine is a general fluorescent sensor for c-di-GMP and 3’,3’-cGAMP. Mol BioSyst.

