# Supplementary material for: Responsive manganese-based nanoplatform amplifying cGAS-STING activation for immunotherapy
Source: Biomater Res. 2023 Apr 15;27:29. doi: 10.1186/s40824-023-00374-x (PMC10105937; doi:10.1186/s40824-023-00374-x)
Supplement: Supplementary file 1 — Supplementary Material 1 [file 40824_2023_374_MOESM1_ESM.pdf]

Supporting Information

Responsive manganese-based nanoplatform amplifying cGAS-STING activation for immunotherapy

Qingbin He,[1,2], Runxiao Zheng[2], Junchi Ma[1], Luyang Zhao[2], Yafang Shi[2],

*Jianfeng Qiu[3]**

1. School of Radiology, Shandong First Medical University & Shandong Academy of Medical Sciences, Tai'an, 271000, China

2. Medical Science and Technology Innovation Center, Shandong First Medical University & Shandong Academy of Medical Sciences, Jinan 250000, China

3. School of Radiology, the Second Affiliated Hospital of Shandong First Medical University, Tai’an, 271016, China

Corresponding author:

Jianfeng Qiu

The Second Affiliated Hospital of Shandong First Medical University, School of Radiology, Shandong First Medical University & Shandong Academy of Medical Sciences, No. 619 Changcheng Road, Tai’an, 271016, China, jfqiu100@163.com

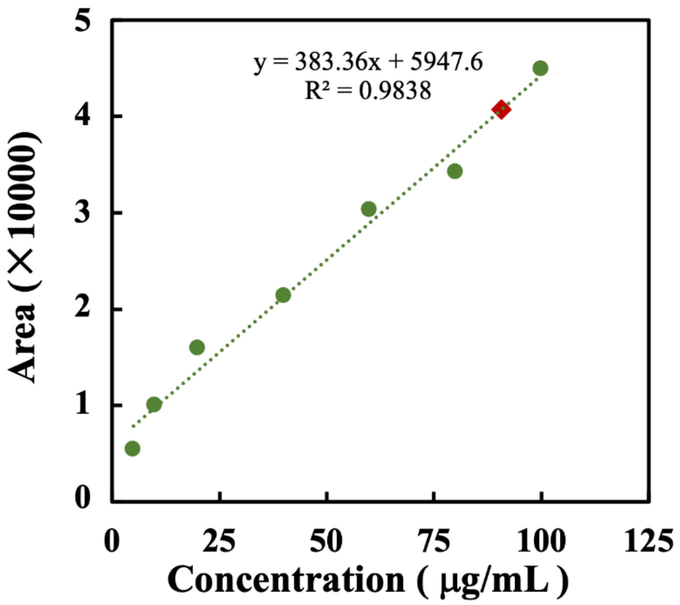

Figure S1. High performance liquid chromatography (HPLC) calibration curve for measuring ZPP loading efficiency. According to the standard curve of ZPP, the mass content of ZPP is 90.77 μg/mL in MPCZ solutions (2 mg/mL), equivalented to 4.54 wt%.

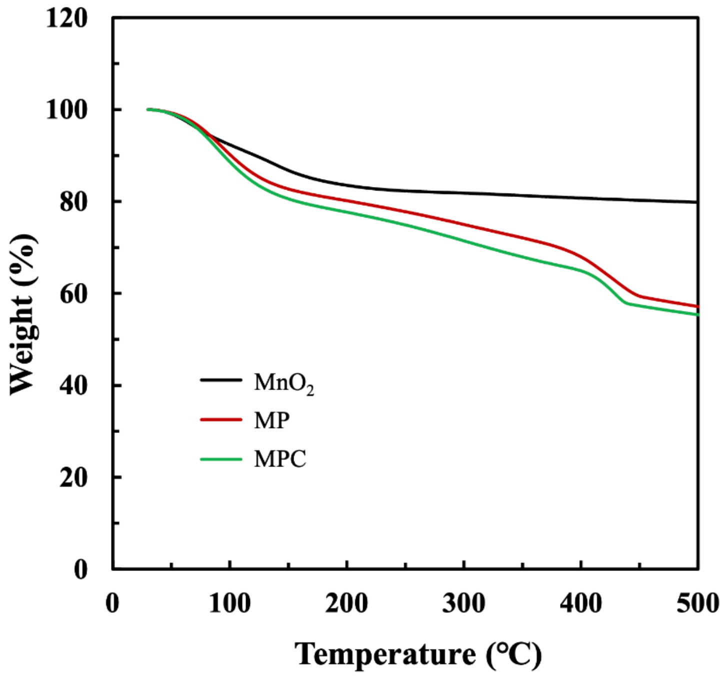

Figure S2. Thermal gravimetric analysis (TGA) of MnO_2, MP NPs and MPC NPs. The mass fractions of PDA and NH_4HCO_3 in MP and MPC can be estimated by matching their mass loss with that of MnO2, respectively. According to the standard curve of ZPP, the mass content of ZPP is 62.5 μg/mg (ZPP/MnO_2). The estimated mass fractions of MnO_2, PDA, NH_4HCO_3, and ZPP in MPCZ are 71.9%, 21.3%, 2.1%, and 4.7%, respectively, based on the measurements mentioned above.

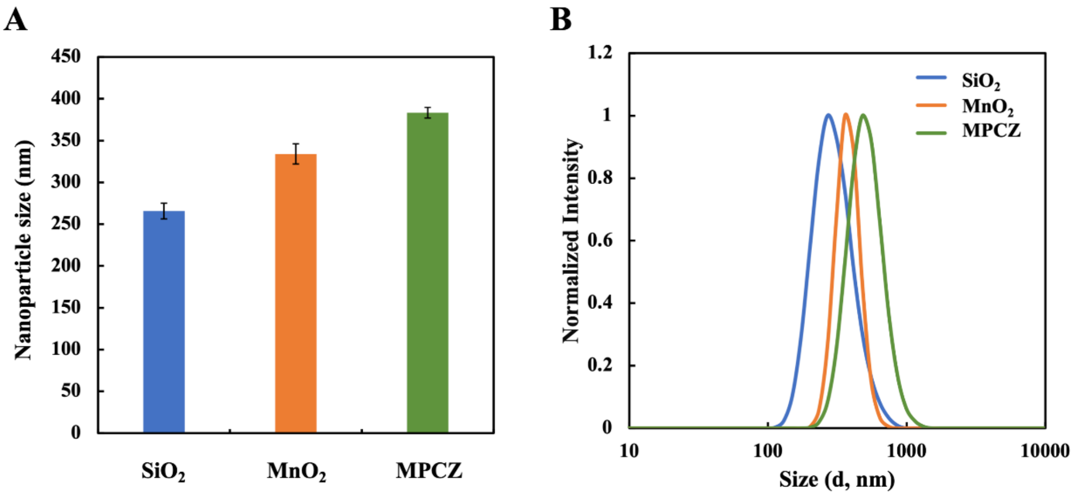

Figure S3. DLS characterization (A) hydrodynamic sizes and (B) size distribution of SiO_2, $hMnO_2$ and MPCZ NPs in H_2O.

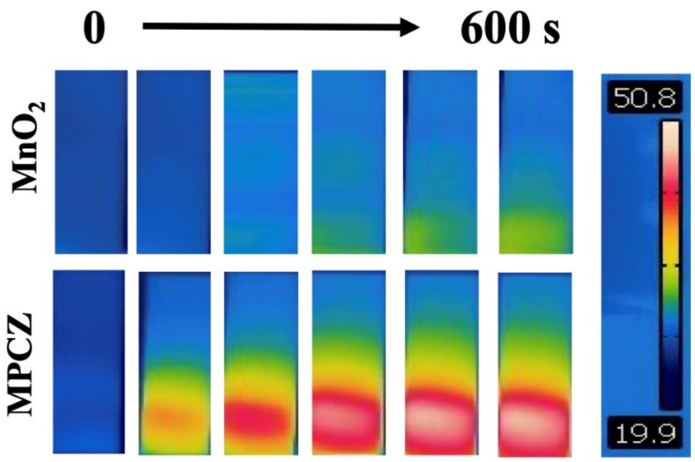

Figure S4. Infrared thermal images of MPCZ (400 μg/mL) and MnO_2 (400 μg/mL).

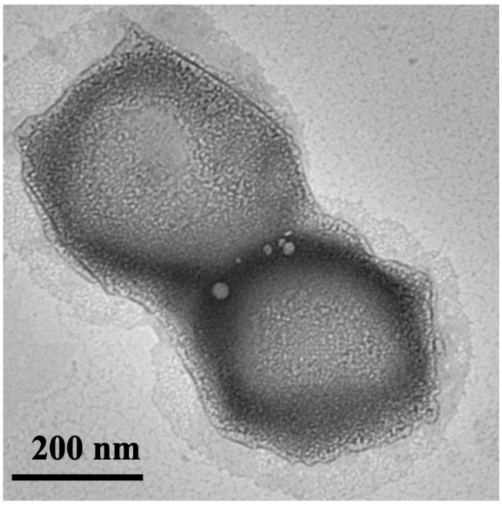

Figure S5. The TEM images of MPCZ NPs under NIR laser irradiation (1 W/cm^2, 10 min).

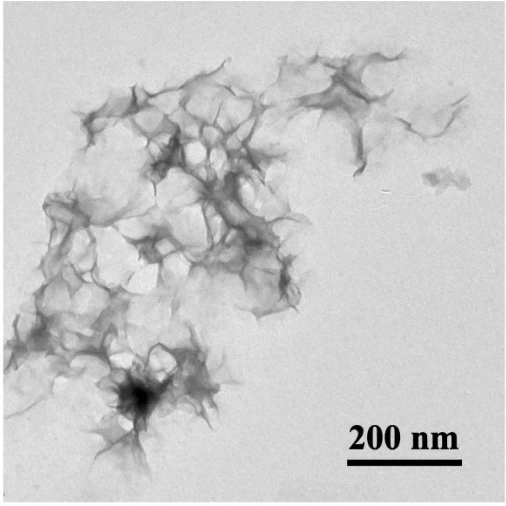

Figure S6. TEM images of MPCZ NPs after incubation in PBS solution (pH = 6.5) with 5 mM GSH under NIR laser irradiation (1 W/cm^2, 10 min).

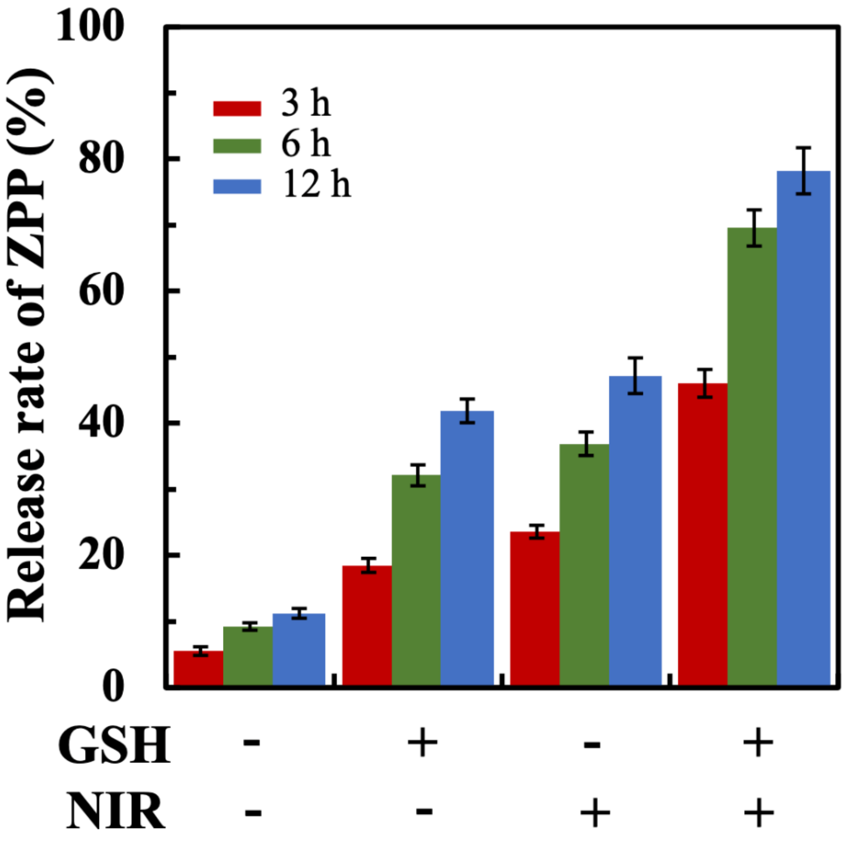

Figure S7. Percentages of released ZPP from MPCZ NPs over time with presence or absence of GSH and NIR laser irradiation according to HPLC.

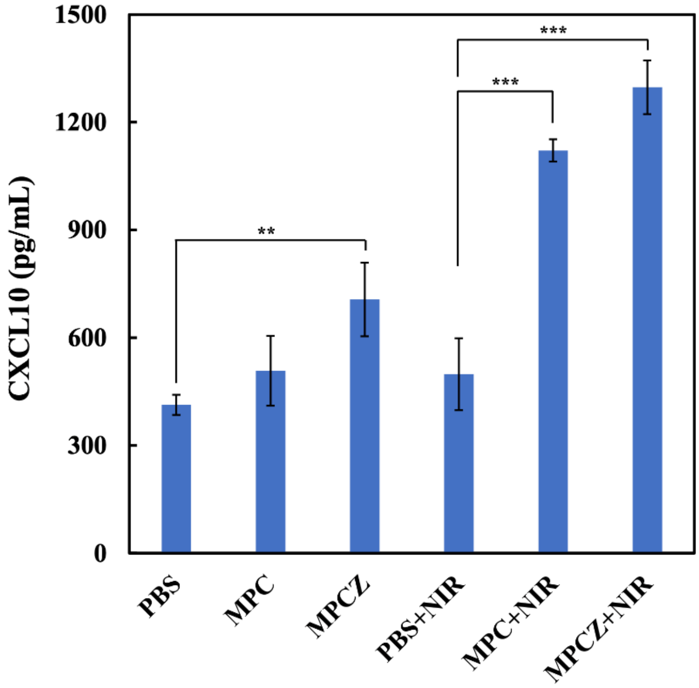

Figure S8. The release of CXCL10 from PBS, MPC NPs and MPCZ NPs treated 4T1 cells with or without NIR laser irradiation detected by ELISA kit (n=3). Labeled asterisk represents statistical significance compared with PBS group via one-way ANOVA with the Tukey post-hoc test. ** $p < 0.01$, *** $p < 0.001$.

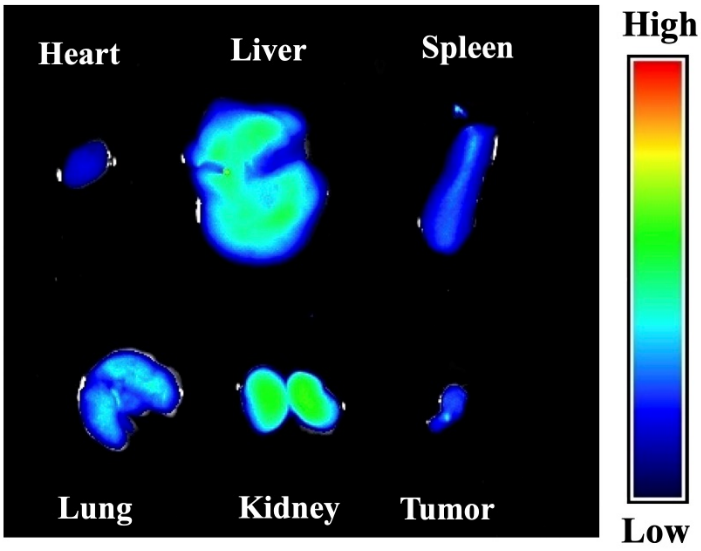

Figure S9. Fluorescence images of main organs and tumors at 24 h post-injection of free Cy5.

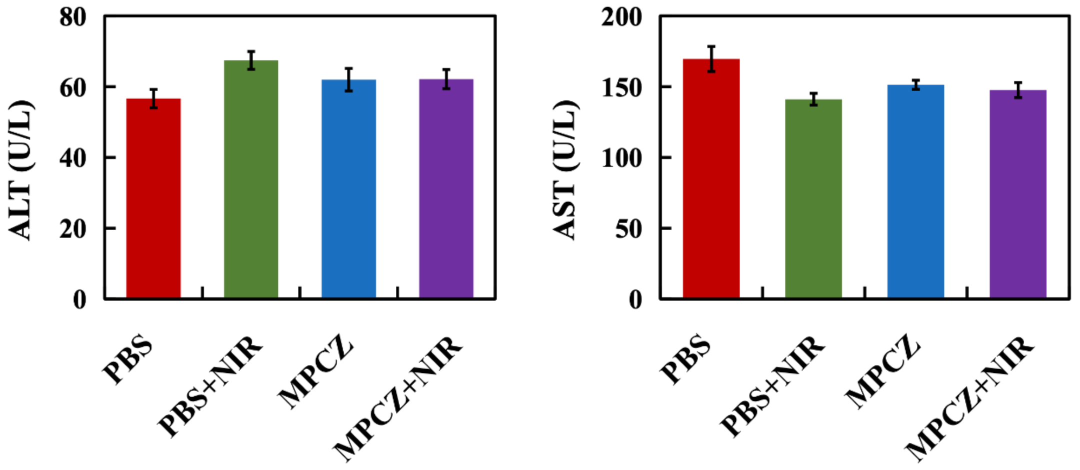

Figure S10. Blood biochemical analysis of liver function. AST, aspartate transferase; ALT, alanine transferase.

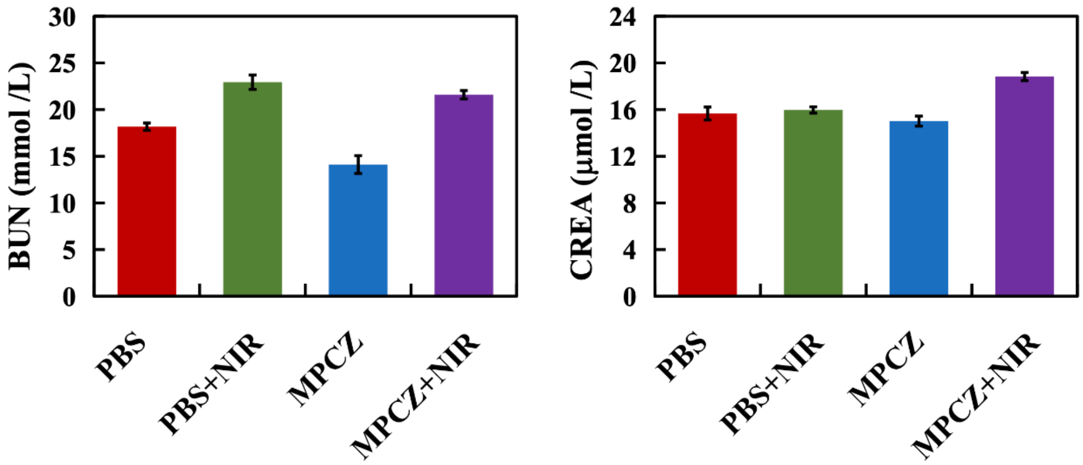

Figure S11. Blood biochemical analysis of kidney function. BUN, blood urea nitrogen; CREA, creatinine.

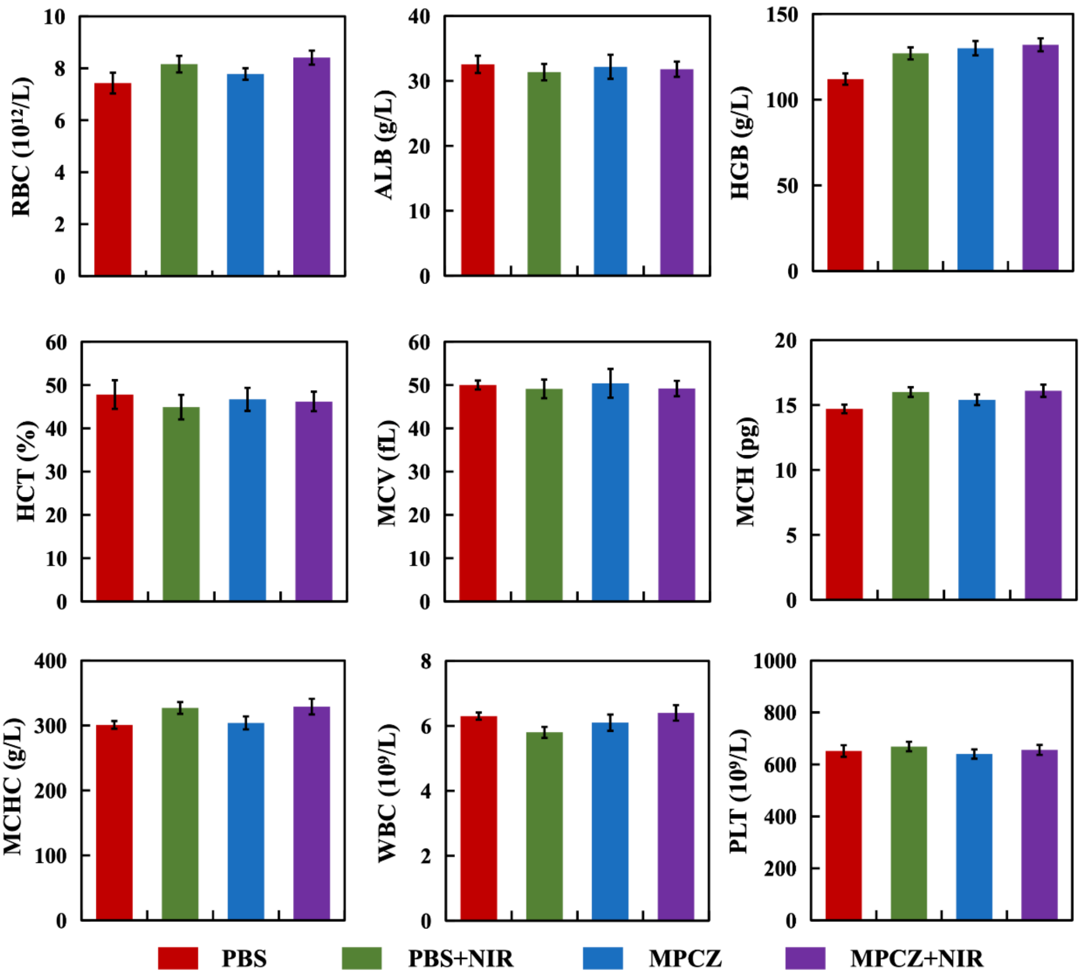

Figure S12. Blood hematological analysis of PBS and MPCZ NPs (with or without NIR) 14 days post-injection. RBC, red blood cells; ALB, serum albumin; HGB, hemoglobin; HCT, hematocrit; MCV, mean capsular volume; MCH, mean capsular hemoglobin; MCHC, mean capsular hemoglobin concentration; WBC, white blood cells, PLT, platelets.

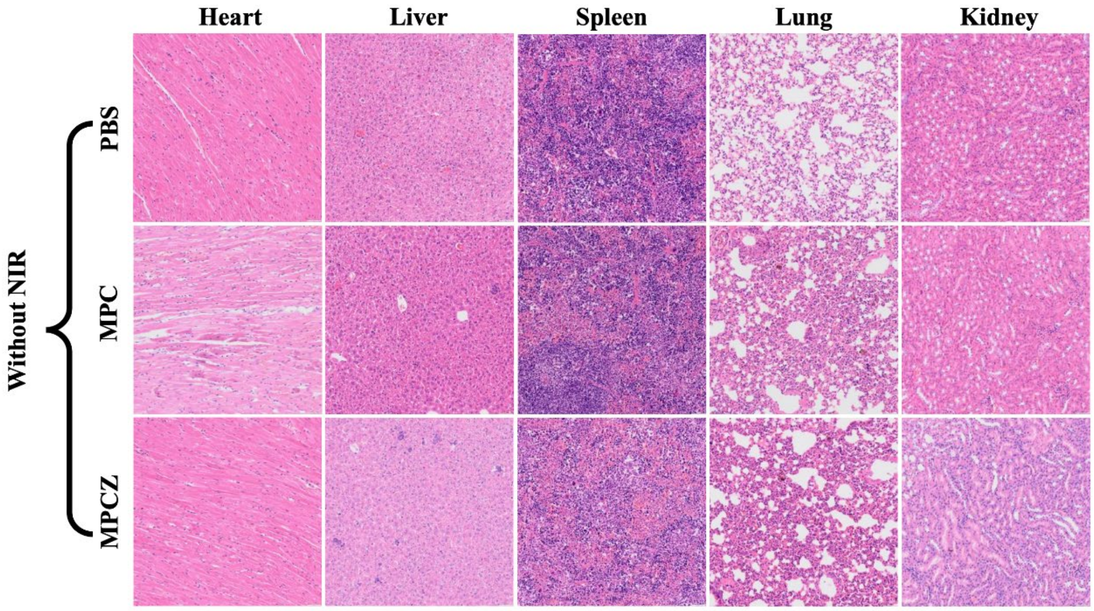

Figure S13. H&E staining of major organs from different groups without NIR irradiation. The mice were intravenously administered PBS, MPC, MPCZ (comparable to 20 mg/kg mouse for MPC and 1 mg/kg mouse for ZPP). After 14 days of treatment, the major organs (heart, liver, spleen, lung and kidney) of different groups of mice were obtained for H&E staining.

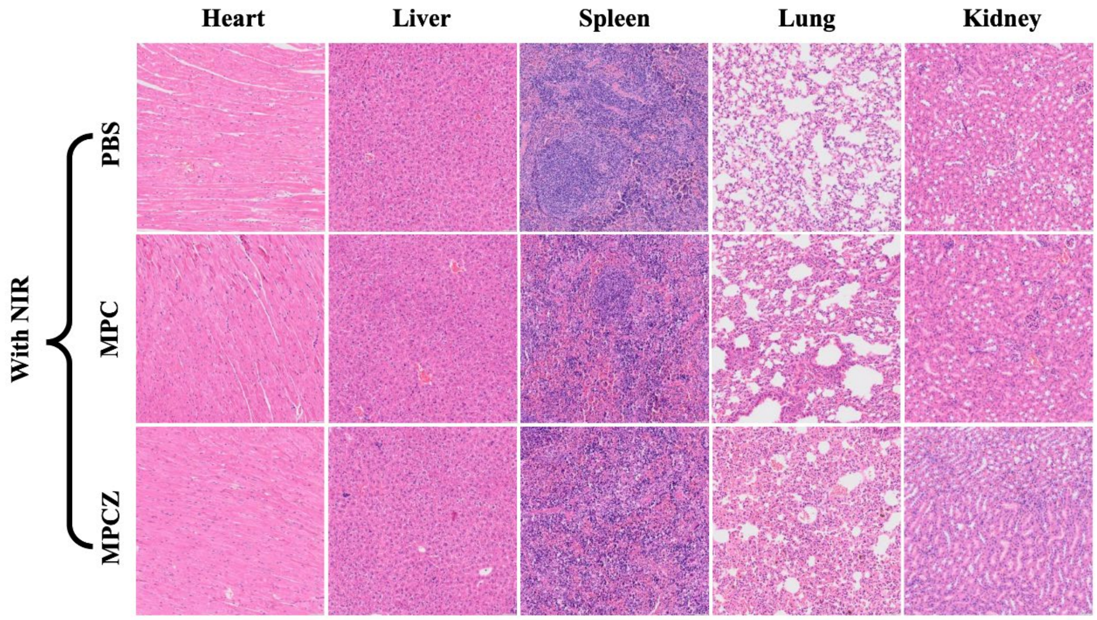

Figure S14. H&E staining of major organs from different groups with NIR laser irradiation. The mice were intravenously administered PBS, MPC, MPCZ (comparable to 20 mg/kg mouse for MPC and 1 mg/kg mouse for ZPP). After 24 h, the tumor region was irradiated under NIR laser irradiation of 1 W/cm^2 for 10 min. After 14 days of treatment, the major organs (heart, liver, spleen, lung and kidney) of different groups of mice were obtained for H&E staining.

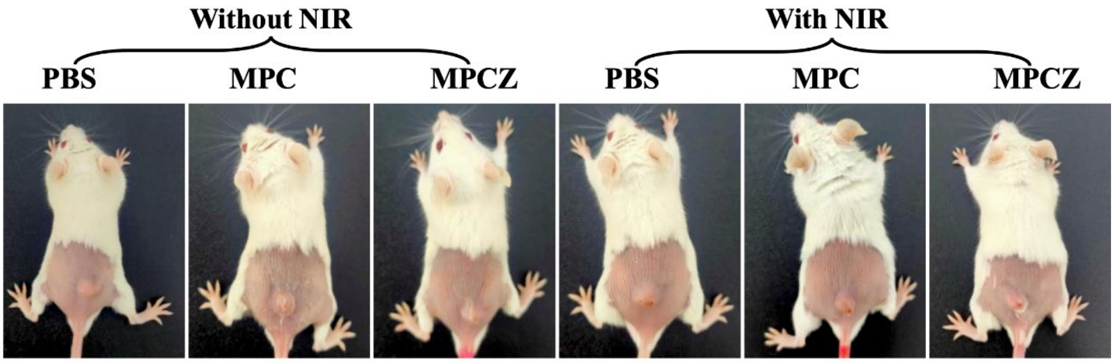

Figure S15. Representative photographs of mice at the beginning of treatment.

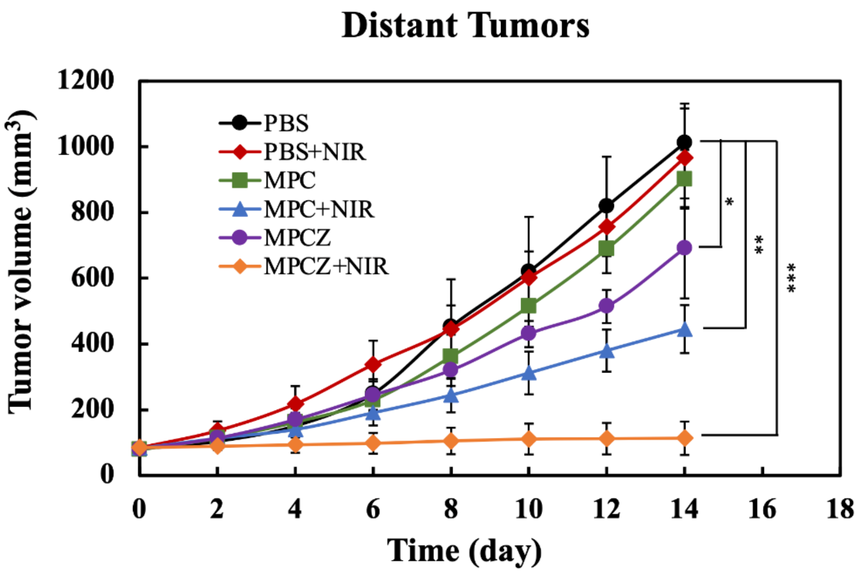

Figure S16. Tumor growth curves of mice for the abscopal tumors assay (n = 5, mean ± SD).

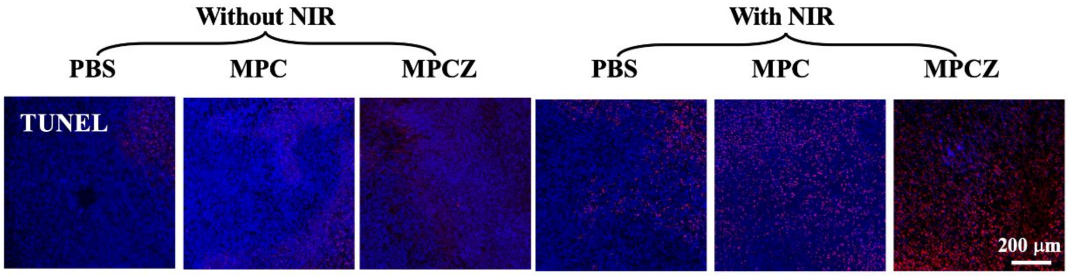

Figure S17. Tumor tissue staining by TUNEL after 14 days of treatments.

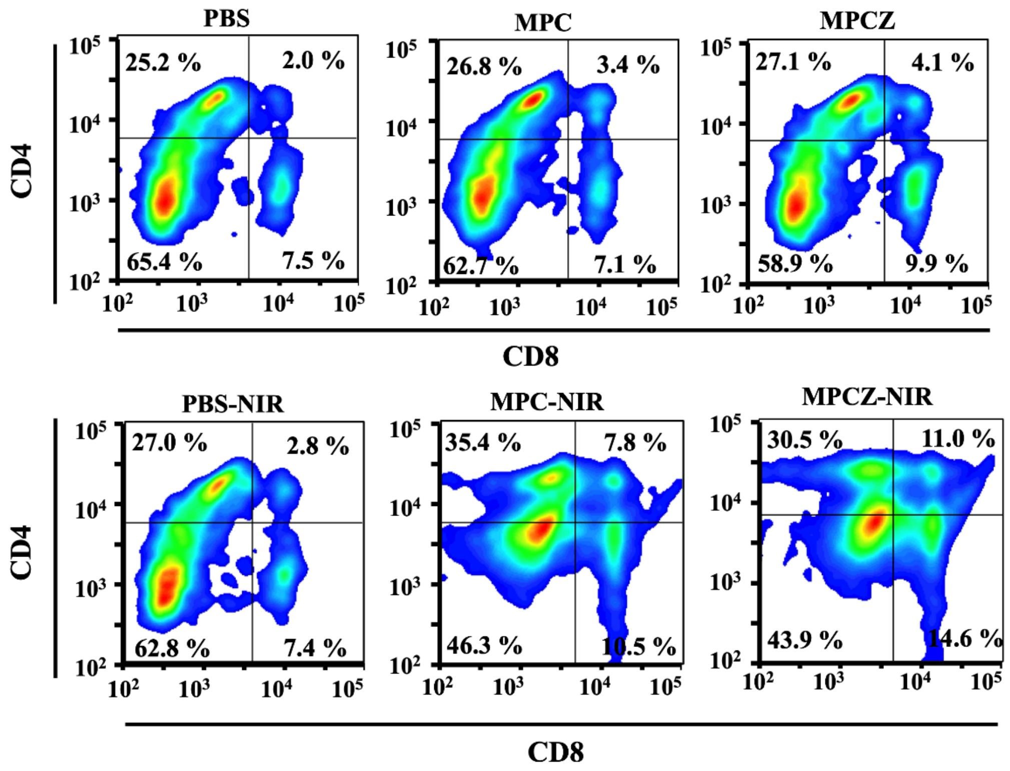

Figure S18. Flow cytometric analysis for evaluating CD4$^+$ and CD8$^+$ T lymphocytes in spleen.

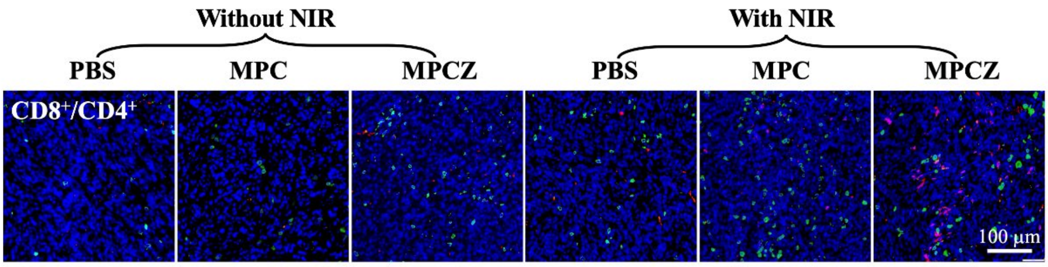

Figure S19. Fluorescence staining of $CD4^+$ or $CD8^+$ T lymphocytes in tumor tissues after 14 days of treatments.
